# LncRNA LIMp27 Regulates the DNA Damage Response through p27 in p53‐Defective Cancer Cells

**DOI:** 10.1002/advs.202204599

**Published:** 2023-01-13

**Authors:** Ting La, Song Chen, Xiao Hong Zhao, Shuai Zhou, Ran Xu, Liu Teng, Yuan Yuan Zhang, Kaihong Ye, Liang Xu, Tao Guo, Muhammad Fairuz Jamaluddin, Yu Chen Feng, Hai Jie Tang, Yanliang Wang, Qin Xu, Yue Gu, Huixia Cao, Tao Liu, Rick F. Thorne, Feng‐Min Shao, Xu Dong Zhang, Lei Jin

**Affiliations:** ^1^ Translational Research Institute Henan Provincial and Zhengzhou City Key laboratory of Non‐coding RNA and Cancer Metabolism Henan International Join Laboratory of Non‐coding RNA and Metabolism in Cancer Henan Provincial People's Hospital Academy of Medical Sciences Zhengzhou University Zhengzhou Henan 450053 China; ^2^ Noncoding Cancer Biomarkers and Therapeutics Group Cancer Detection & Therapy Research Program Hunter Medical Research Institute Callaghan New South Wales 2305 Australia; ^3^ School of Biomedical Sciences and Pharmacy The University of Newcastle Callaghan New South Wales 2308 Australia; ^4^ National‐Local Joint Engineering Research Center of Biodiagnosis & Biotherapy The Second Affiliated Hospital Xi'an Jiaotong University Xi'an Shaanxi 710004 China; ^5^ Institute of Medicinal Biotechnology Jiangsu College of Nursing Huai'an Jiangsu 223300 China; ^6^ Institute of Future Agriculture Northwest A&F University Yangling Shaanxi 712100 China; ^7^ School of Medicine and Public Health The University of Newcastle Callaghan New South Wales 2308 Australia; ^8^ Department of Nephrology Henan Provincial Key Laboratory of Kidney Disease and Immunology Henan Provincial Clinical Research Center for Kidney Disease Henan Provincial People's Hospital Zhengzhou Henan 450053 China; ^9^ Children's Cancer Institute Australia for Medical Research University of New South Wales Sydney New South Wales 2750 Australia

**Keywords:** colon cancer, E2F1, hnRNPA0, lncRNA, LIMp27, p27

## Abstract

P53 inactivation occurs in about 50% of human cancers, where p53‐driven p21 activity is devoid and p27 becomes essential for the establishment of the G1/S checkpoint upon DNA damage. Here, this work shows that the E2F1‐responsive lncRNA LIMp27 selectively represses p27 expression and contributes to proliferation, tumorigenicity, and treatment resistance in p53‐defective colon adenocarcinoma (COAD) cells. LIMp27 competes with p27 mRNA for binding to cytoplasmically localized hnRNA0, which otherwise stabilizes p27 mRNA leading to cell cycle arrest at the G0/G1 phase. In response to DNA damage, LIMp27 is upregulated in both wild‐type and p53‐mutant COAD cells, whereas cytoplasmic hnRNPA0 is only increased in p53‐mutant COAD cells due to translocation from the nucleus. Moreover, high LIMp27 expression is associated with poor survival of p53‐mutant but not wild‐type p53 COAD patients. These results uncover an lncRNA mechanism that promotes p53‐defective cancer pathogenesis and suggest that LIMp27 may constitute a target for the treatment of such cancers.

## Graphical abstract

P53 inactivation occurs in ≈50% of human cancers, and p53‐deficient cancer cells are often resistant to chemotherapy‐induced apoptosis. Here, this work uncovers a long nonprotein‐coding RNA LIMp27 mechanism that promotes the pathogenesis of p53‐deficient cancers and suggest that targeting LIMp27 alone or in combination with chemotherapy may benefit patients with such cancers.

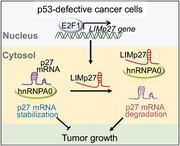

## 1 Introduction

Cells constantly activate the DNA damage response (DDR) in response to sustained DNA damage caused by metabolic activities and environmental insults, such as radiation and thermal disruption. The DDR represents a multifaceted array of biological processes that identifies and repairs DNA damage, in turn enabling cell survival and proliferation.^[^ ^1^, ^2^ ^]^ An important DDR component is the guardian of the genome, the tumor suppressor p53.^[^ ^3^ ^]^ Following DNA damage, p53 functions to arrest cell cycle progression at the G0/G1 phase, primarily through transcriptional activation of the cyclin‐dependent kinase (CDK) inhibitor p21, allowing sufficient time for DNA repair.^[^ ^4^, ^5^, ^6^ ^]^ However, after excessive DNA damage and/or when repair is compromised, p53 activates pro‐apoptotic genes, such as PUMA and NOXA, eliminating the injured cells through apoptosis.^[^ ^4^, ^7^ ^]^ When normal repair fails and apoptosis does not occur, repair errors cause accumulative genetic mutations that drive malignant transformation.^[^ ^8^, ^9^ ^]^

Cancer cells are more prone to DNA damage because of increased metabolic activities driven by oncogenic signaling and the often‐adverse microenvironment.^[^ ^10^ ^]^ However, p53 is lost or mutated in ~50% of human cancers.^[^ ^3^ ^]^ This is not only involved in cancer development and progression, but also responsible for the resistance to therapeutics that exerts their effects through causing damage to DNA, such as platinum‐based drugs and ionizing radiation.^[^ ^4^ ^]^ In p53‐defective cancer cells, the other major CDK inhibitor p27 plays a decisive role in the establishment of the G1/S checkpoint.^[^ ^11^ ^]^ Nevertheless, the outcome of this function of p27 appears paradoxical in the cancer context.^[^ ^12^ ^]^ On the one hand, it impedes cell cycle progression and thus conceivably slows down tumor growth in vivo.^[^ ^13^ ^]^ On the other hand, it enables tumor cells to evade the cytotoxic effects of DNA‐damaging therapeutics.^[^ ^14^ ^]^ Indeed, while p27 levels are reduced in many cancer types and this is associated with poor patient outcomes,^[^ ^12^ ^]^ low p27 expression has also been documented to correlate with better responses to DNA‐damaging drugs.^[^ ^12^ ^]^

The expression of p27 is primarily controlled by post‐translational mechanisms.^[^ ^15^ ^]^ Nevertheless, several transcription factors including forkhead box class O family (FoxO) proteins and E2F1 can transcriptionally activate CDKN1B, the gene encoding p27.^[^ ^16^ ^]^ Furthermore, p27 mRNA can be stabilized by cytoplasmically localized heterogeneous ribonucleoprotein A0 (hnRNPA0), a key process that upregulates p27 expression in p53‐defective cancer cells upon DNA damage.^[^ ^11^, ^17^ ^]^ Other important, but arguably less well appreciated mechanisms that regulate p27 expression involve noncoding RNAs.^[^ ^18^ ^]^ For example, miRNA‐221/‐222 and miRNA‐455‐3p/‐27b‐3p regulate CDKN1B transcription and p27 stabilization, respectively.^[^ ^19^, ^20^ ^]^ whereas the long noncoding RNAs (lncRNAs) OVAAL and TRMP suppress internal ribosome entry site (IRES)‐dependent p27 translation, and the shorter variant of TRMP, TRMP‐S represses p27 expression through both transcriptional and posttranslational mechanisms.^[^ ^21^, ^22^ ^]^

Here we present evidence that the lncRNA LIMp27 selectively represses p27 expression and contributes to the increased proliferation, tumorigenicity, and resistance to DNA‐damaging therapeutics in p53‐mutant colon adenocarcinoma (COAD) cells. Moreover, we show that LIMp27 executes this function through competing with p27 mRNA for binding to cytoplasmic hnRNPA0. We also demonstrate that LIMp27 is frequently upregulated in COAD tissues through E2F1‐mediated transcriptional activation, and that its high expression is associated with poor outcomes in patients with mutant p53‐expressing tumors. Collectively, these findings suggest that interference with LIMp27, alone or in combination with DNA‐damaging therapeutics, represents a potential approach for the treatment of p53‐defective cancers.

## 2 Results

### 2.1 Identification of LIMp27 as an E2F1‐responsive lncRNA that Selectively Supports p53‐defective Cancer Cells

**Figure 1:**
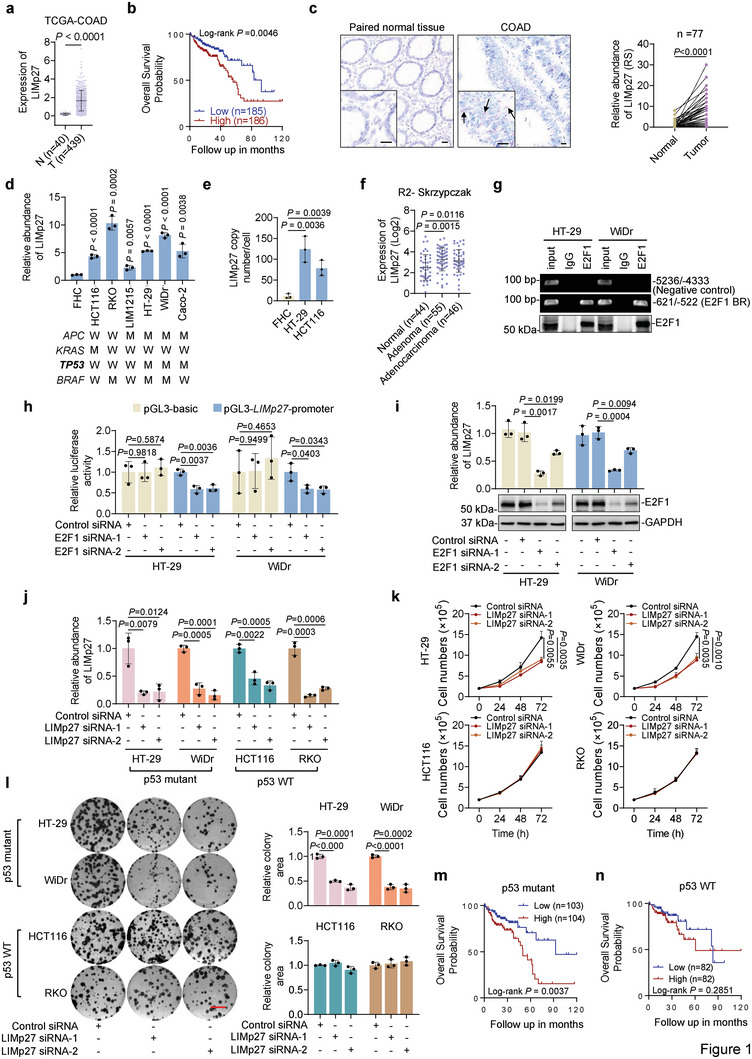
Identification of LIMp27 as an E2F1‐responsive lncRNA that selectively supports the viability of p53‐defective cancer cells. a) LIMp27 is upregulated in COAD compared with corresponding normal tissues as revealed by analysis of the lncRNA expression data in the TCGA dataset. Data are mean ± s.d.; two‐tailed Student's *t*‐test. N: normal tissues; T: tumor tissues. b) Kaplan–Meier analysis of the probability of overall survival of COAD patients (*n* = 371) derived from the TCGA using the median of LIMp27 levels as the cut‐off. c) Representative microphotographs and quantitation of in situ hybridization (ISH) analysis of LIMp27 expression in formalin‐fixed paraffin‐embedded (FFPE) COAD tissues (*n* = 77 biologically independent samples) compared with corresponding paired adjacent normal tissues. Scale bar, 20 µm. RS: reactive score. Two‐tailed Student's *t*‐test. d) qPCR analysis showing that LIMp27 was generally more abundant in colon cancer cell lines than in the normal colon epithelial cell line FHC. Data are mean ± s.d.; *n* = 3 independent experiments, two‐tailed Student's *t*‐test. e) Absolute quantitation of LIMp27 in HT‐29 and HCT116 COAD cell lines and the normal colon epithelial cell line FHC using qPCR. Data are mean ± s.d.; *n* = 3 independent experiments, two‐tailed Student's *t*‐test. f) Comparison of LIMp27 expression between normal colon mucosa, colon adenoma, and colon cancer tissues derived from R2 public dataset. Data are mean ± s.d.; one‐way ANOVA followed by Tukey's multiple comparisons test. g) Chromatin immunoprecipitation (ChIP) analysis of the association between endogenous E2F1 and the E2F1‐binding motifs at the promoter of LIMp27 in HT‐29 and WiDr cell lines. Data are representatives of three independent experiments. h) SiRNA knockdown of E2F1 reduced the transcriptional activity of a LIMp27 promoter reporter construct (pGL3‐LIMp27 promoter). Data are mean ± s.d.; *n* = 3 independent experiments, two‐tailed Student's *t*‐test. i) E2F1 silencing downregulated LIMp27 expression in HT‐29 and WiDr cell lines. Data are representatives or mean ± s.d.; *n* = 3 independent experiments, one‐way ANOVA followed by Tukey's multiple comparisons test. j–l) SiRNA knockdown of LIMp27 (j) inhibited cell proliferation (k) and clonogenicity (l) in HT‐29 and WiDr (p53 mutant) but not in HCT116 and RKO (p53 WT) cell lines. Data are mean ± s.d. or representatives; *n* = 3 independent experiments, one‐way ANOVA followed by Tukey's multiple comparison test. Scale bar, 1 cm. m,n) Kaplan–Meier analysis of the probability of overall survival of COAD patients with tumors carrying mutant p53 (m) and wild‐type p53 tumors (n) derived from the TCGA using the median of LIMp27 levels as the cut‐off.

Through interrogating the RNA‐sequencing (RNA‐seq) COAD dataset acquired from the Cancer Genome Atlas (TCGA),^[^ ^23^ ^]^ we identified a panel of lncRNAs that were commonly upregulated in >80% of COAD samples compared with normal colon tissues (Figure S1a, Supporting Information).^[^ ^24^, ^25^ ^]^ The top two ranked lncRNAs were MILIP [long noncoding RNA inactivating P53; also known as MAFG‐AS1 (v‐maf avian musculoaponeurotic fibrosarcoma oncogene homolog G antisense RNA1)], which is known to promote COAD pathogenesis through p53 repression,^[^ ^25^ ^]^ and LINC01356 that we now call LIMp27 (Long noncoding RNA Inhibiting the mRNA of p27) given its inhibitory effect on p27 mRNA expression (see below) (**Figure** **1**a). Instructively, akin to the high expression of MILIP,^[^ ^25^ ^]^ high LIMp27 expression was associated with poor overall patient survival (OS) (Figure 1b). On this basis, we investigated the potential role of LIMp27 in COAD pathogenesis.

Using in situ hybridization (ISH), we first independently confirmed that LIMp27 expression was frequently upregulated in a cohort of formalin‐fixed paraffin‐embedded (FFPE) tissues comparing COAD samples with paired adjacent normal colon epithelial tissues (Figure 1c, Table S1, Supporting Information). Similarly, LIMp27 was commonly expressed at higher levels in COAD cell lines than in the normal colon epithelial cell line FHC, irrespective of their mutational status in APC, KRAS, TP53 and BRAF, the most common genetic anomalies in COAD (Figure 1d). Absolute quantitation showed that there were ≈ 124 and ≈ 78 LIMp27 molecules per HT‐29 and HCT116 COAD cell, respectively, compared with ≈11 LIMp27 molecules per FHC cell (Figure 1e). Of note, LIMp27 levels did not differ among different pathological stages of COAD (Table S1, Supporting Information). Likewise, there were no significant differences in LIMp27 expression between COAD groups stratified by tumor grade, patient gender, nor their median age at diagnosis (Table S1, Supporting Information). Furthermore, no significant differences were found in LIMp27 expression between COAD and colon adenomas (pre‐neoplastic colon lesions), whereas LIMp27 expression was upregulated in colon adenomas compared with normal colon epithelia (Figure 1f), suggesting that LIMp27 upregulation is commonly an early event during COAD development.

To understand how LIMp27 is upregulated in COAD cells, we interrogated its gene promoter using the JASPAR CORE database (jsapar.genereg.net) and found potential binding sites for 538 transcription factors (TFs). SiRNA screening of the top 8 TFs (TFAP4, ETV4, POU5F1B, MLXIPL, MYC, TGIF2, E2F1, ZBTB12) that were significantly upregulated with expression levels being correlated with LIMp27 expression in COADs revealed that knockdown of E2F1 but not the other 7 TFs significantly suppressed the expression of LIMp27 (Figure S1b, Supporting Information). The predicted E2F1‐binding motif was located to the −603/−596 region (E2F1‐BR) of the proximal promoter of the LIMp27 gene (Figure S1c, Supporting Information). Indeed, co‐precipitation was observed between E2F1 and the LIMp27 promoter and their interaction was required for the transcriptional upregulation of LIMp27 as revealed in reporter assays showing that LIMp27 transcriptional activity was inhibited when the E2F1‐BR was deleted (Figure 1g, Figure S1d, Supporting Information). Moreover, co‐transfection of E2F1 selectively enhanced the transcriptional activity of the LIMp27 reporter whereas knockdown of E2F1 diminished reporter activity (Figure 1h, Figure S1e, Supporting Information). Consistently, knockdown of E2F1 reduced, whereas overexpression of E2F1 increased, endogenous LIMp27 expression in HT‐29 and HCT116 cells, along with the lncRNA PLANE known to be transcriptionally regulated by E2F1 (Figure 1i, Figure S1f–h, Supporting Information).^[^ ^26^ ^]^ Therefore, LIMp27 is transcriptionally activated by E2F1 through the identified E2F1‐BR in COAD cells. Consistent with this mechanism, a general trend was noted in COAD tissues where LIMp27 levels correlated with E2F1 mRNA expression levels (Figure S1i, Supporting Information). Moreover, qPCR analysis of a cohort of freshly isolated COAD samples showed that the levels of LIMp27 were indeed correlated with E2F1 expression levels (Figure S1j, Supporting Information).

The gene encoding LIMp27 is located head‐to‐head and shares a common region with the gene encoding another lncRNA, LINC01357, on chromosome 1p13.2 (Figure S1k, Supporting Information). Nevertheless, neither knockdown nor overexpression of LIMp27 impinged upon LINC01357 expression, and similarly, either knockdown or overexpression of LINC01357 did not affect the expression of LIMp27 (Figure S1l–o, Supporting Information). Together this indicates that no regulatory interplay occurs between the two neighboring lncRNAs genes. In support, although LINC01357 expression was similarly increased in COAD compared with normal colon tissues, its expression was not associated with poor patient survival as was evident with LIMp27 expression (Figure S1p,q, Supporting Information).^[^ ^27^ ^]^ Moreover, in contrast to the regulation of LIMp27 by E2F1 (Figure 1i, Figure S1g, Supporting Information), knockdown or overexpression of E2F1 in HT‐29 and HCT116 cells did not alter the expression of LINC01357 (Figure S1r,s, Supporting Information), suggesting that despite their close proximity, E2F1 selectively transactivates LIMp27 but not LINC01357.

Of the three annotated LIMp27 isoforms (LIMp27‐201, LIMp27‐202, and LIMp27‐203; Vega Genome Browser), LIMp27‐202 was markedly more abundant than others in multiple COAD cell lines, including HT‐29, WiDr, HCT116, RKO and Caco‐2, as shown in RT‐PCR analysis with isoform‐specific primers (Figure S2a,b, Supporting Information). The LIMp27‐202 isoform consists of 4 exons (E1 – E4) with minimum free energy modeling predicting a broadly tripod‐like structure with E4 contributing to two poles and E2 and E3 contributing to one pole, whereas E1 disperses within one of the two poles formed by E4 and the pole constituted by E2 and E3 (Figure S2c, Supporting Information).

We sought to gain insights into the potential function of LIMp27 in diverse types of cancer cell lines, including the COAD cell lines HT‐29, WiDr, HCT116 and RKO, breast cancer cell lines MDA‐MB‐231 and MCF‐7, and non‐small cell lung cancer cell lines H226 and A549. Strikingly, siRNA knockdown of LIMp27 markedly reduced the viability and clonogenicity in HT‐29, WiDr, MDA‐MB‐231, and H226 cells that harbored mutant p53, but not in wild‐type p53‐expressing HCT116, RKO, MCF‐7, and A549 cells (Figure 1j–l, Figure S2d–f, Supporting Information). In accordance with this finding, the overexpression of LIMp27 promoted, albeit moderately, the viability and clonogenicity in HT‐29 and WiDr but not HCT116 and RKO COAD cells (Figure S2g,h, Supporting Information). Extended analyses confirmed the inhibitory effect of LIMp27 knockdown in Caco‐2 and SW480 cells harboring mutant p53 (Figure S2i, Supporting Information). Consistently, similar to HCT116 and RKO cells, LIMp27 knockdown in LIM1215 cells which express wild‐type p53 did not affect their viability (Figure S2j, Supporting Information). Moreover, when COAD patients in the TCGA dataset were stratified according to their TP53 mutational status, it appeared that high LIMp27 expression was more closely associated with poor OS of patients with tumors carrying mutant TP53 (Figure 1m), whereas there was no significant relationship between LIMp27 expression and OS of patients with wild‐type TP53 tumors (Figure 1n). Collectively these data suggest that although its expression is commonly upregulated in COAD, LIMp27 selectively promotes cell viability in p53‐defective COAD.

To substantiate this notion, we compared the effects of combinatorial knockdown of p53 and LIMp27 in wild‐type and mutant p53 COAD cell lines. On the one hand, knockdown of p53 rendered wild‐type p53 HCT116 and RKO cells susceptible, albeit moderately, to inhibition of cell viability by LIMp27 knockdown (Figure S2k, Supporting Information). On the other hand, knockdown of mutant p53 in p53‐mutant HT‐29 and WiDr cells did not impinge on the inhibition of cell viability caused by LIMp27 knockdown (Figure S2l, Supporting Information). Taken together, these data provide strong evidence that the promotion of cell viability mediated by LIMp27 in p53‐defective COAD is not directly mediated by mutant p53.

### 2.2 LIMp27 Promotes p53‐Mutant COAD Cell Proliferation and Tumorigenicity

**Figure 2:**
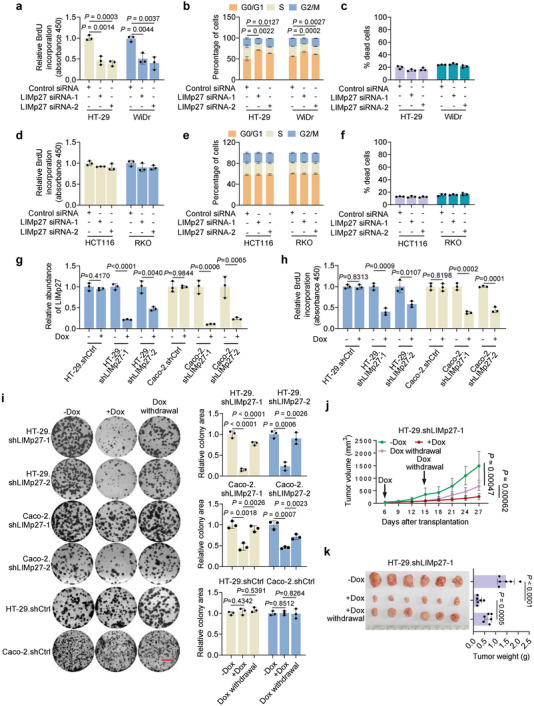
LIMp27 promotes p53‐mutant COAD cell proliferation and tumorigenicity. a–c) SiRNA knockdown of LIMp27 inhibited 5‐bromo‐2′‐deoxyuridine (BrdU) incorporation (a) and caused G0/G1 phase cell cycle arrest (b) but did not cause significant cell death (c) in HT‐29 and WiDr cell lines. Data are mean ± s.d; *n* = 3 independent experiments, one‐way ANOVA followed by Tukey's multiple comparison test. d–f, SiRNA knockdown of LIMp27 did not affect BrdU incorporation (d), G0/G1 phase cell cycle arrest (e), and cell death (f) in HCT116 and RKO cell lines. Data are mean ± s.d; *n* = 3 independent experiments, one‐way ANOVA followed by Tukey's multiple comparison test. g,h) Induced knockdown of LIMp27 by doxycycline (Dox, 1 µg mL^−1^) (g) inhibited BrdU incorporation (h) in HT‐29.shLIMp27 and Caco‐2.shLIMp27 cell sublines, but not in HT‐29.shCtrl and Caco‐2.shCtrl cell sublines. Data are mean ± s.d; *n* = 3 independent experiments, two‐tailed Student's *t*‐test. i) Induced knockdown of LIMp27 inhibited the clonogenicity, which was partially reversed by Dox (1 µg mL^−1^) withdrawal of HT‐29.shLIMp27 and Caco‐2.shLIMp27 cell sublines. Similar effects were not observed in HT‐29.shCtrl and Caco‐2.shCtrl cell sublines. Data are representatives or mean ± s.d.; *n* = 3 independent experiments, one‐way ANOVA followed by Tukey's multiple comparisons test. Scale bar, 1 cm. j,k) Growth curves (j) and representative photographs and tumor weights (k) showing induced knockdown of LIMp27 by Dox retarded HT‐29.shLIMp27 xenograft growth, which was reversed by Dox withdrawal in nu/nu mice. Data are mean ± s.d.; *n* = 6 mice per group, one‐way ANOVA followed by Tukey's multiple comparison test. Dox: 2 mg mL^−1^ supplemented with 10 mg mL^−1^ sucrose in drinking water.

We focused on evaluating the mechanism whereby LIMp27 supports the viability of p53‐mutant COAD cells (Figure 1j–l). Notably, LIMp27 knockdown induced marked inhibition of BrdU incorporation and G0/G1 cell cycle arrest but did not cause significant cell death in HT‐29 and WiDr cells (**Figure** **2**a–c), indicative that LIMp27 predominantly promotes cell proliferation in p53‐mutant COAD cells. Consistently, overexpression of LIMp27 caused, albeit moderately, an increase in BrdU incorporation in HT‐29 and WiDr cells (Figure S3a,b, Supporting Information). On the other hand, knockdown or overexpression of LIMp27 did not affect BrdU incorporation and cell cycle progression, nor did it induce cell death in HCT116 and RKO cells (Figure 2d–f, Figures S3c,d, Supporting Information), indicating that the regulatory effect of LIMp27 on cell proliferation is p53‐mutant cell‐specific. Corroborating this finding, LIMp27 knockdown in additional p53‐mutant COAD cell lines induced reductions in BrdU incorporation but not in wild‐type p53 LIM1215 cells (Figure S3e, Supporting Information).

To facilitate further examination of the effects of LIMp27 on COAD growth, particularly in the in vivo setting, we established sublines of p53‐mutant HT‐29 and Caco‐2 cells (HT‐29.shLIMp27 and Caco‐2.shLIMp27, respectively) with conditional LIMp27 knockdown in response to doxycycline (Dox) and corresponding control cell lines (HT‐29.shCtrl and Caco‐2.shLCtrl) (Figure 2g). As anticipated, the addition of Dox in vitro readily inhibited cell proliferation and clonogenicity associated with reduced LIMp27 expression (Figure 2g–i). Moreover, after Dox withdrawal and the recovery of LIMp27 expression, cell clonogenicity was restored, at least partially (Figure 2i). Importantly, the treatment of nu/nu mice bearing tumors established by subcutaneous implantation of HT‐29.shLIMp27 cells but not HT‐29.shCtrl cells with Dox retarded tumor growth (Figure 2j,k, Figure S3f, Supporting Information). This was associated with reduced cell proliferation as shown by decreases in the proportion of Ki‐67‐expressing cells (Figure S3g, Supporting Information). Additionally, the cessation of Dox treatment led to the recovery of LIMp27 expression and tumor regrowth of HT‐29.shLIMp27 cells but not HT‐29.shCtrl cells (Figure 2j,k, Figure S3f, Supporting Information). Together, these results indicate LIMp27 promotes p53‐mutant COAD cell proliferation and tumorigenicity.

### 2.3 LIMp27 Represses p27 Expression through Destabilizing p27 mRNA

**Figure 3:**
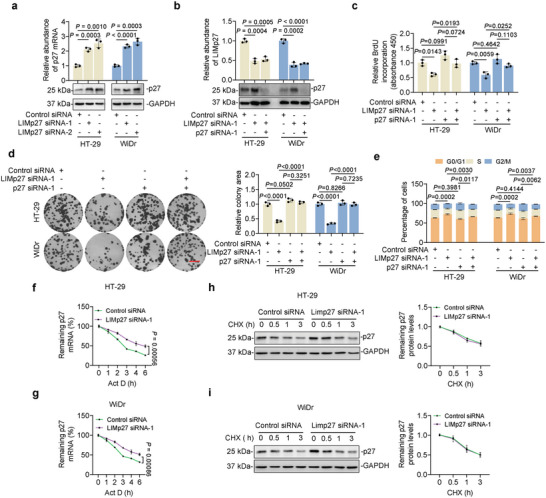
LIMp27 represses p27 expression through destabilizing p27 mRNA. a) LIMp27 knockdown upregulated p27 expression at both mRNA and protein levels. Data are mean ± s.d. or representatives; *n* = 3 independent experiments, one‐way ANOVA followed by Tukey's multiple comparison test. b–e) LIMp27 knockdown‐induced upregulation of p27 expression (b), inhibition of BrdU incorporation (c), and clonogenicity (d), and G0/G1 phase cell cycle arrest (e) were diminished by p27 co‐knockdown in HT‐29 and WiDr cell lines. Data are representatives or mean ± s.d.; *n* = 3 independent experiments, One‐way ANOVA followed by Tukey's multiple comparisons test. Scale bar, 1 cm. f,g) Total RNA from HT‐29 (f) and WiDr (g) cell lines transfected with indicated siRNAs and treated with Actinomycin D (Act D, 1 µg mL^−1^) for indicated periods were subjected to qPCR. Data are mean ± s.d.; *n* = 3 independent experiments, two‐tailed Student's *t*‐test. h,i) Whole‐cell lysates from HT‐29 (h) and WiDr (i) cell lines transfected with indicated siRNAs and treated with cycloheximide (CHX; 5 mg mL^−1^) for indicated periods were subjected to Western blotting (left panel). Quantitation of p27 expression normalized to GAPDH was shown (right panel). Data are representatives or mean ± s.d.; *n* = 3 independent experiments, two‐tailed Student's *t*‐test.

To dissect the mechanism through which LIMp27 promotes p53‐mutant COAD cell proliferation, we compared the transcriptomes of WiDr cells with and without LIMp27 knockdown using two independent siRNAs (Figure S4a, Supporting Information). Gene set enrichment analysis (GSEA) revealed that knockdown of LIMp27 caused downregulation of numerous hallmark_E2F_targets (Figure S4b, Supporting Information), consistent with G0/G1 cell cycle arrest (Figure 2b). Of note, CDKN1B, but not CDKN1A, the gene encoding p21, was increased in cells with LIMp27 knockdown (Figure S4c, Supporting Information). These differential effects of LIMp27 knockdown on CDKN1A and CDKN1B were readily confirmed using qPCR analysis in WiDr and other p53‐mutant COAD cell lines (**Figure** **3**a, Figure S4d, Supporting Information). Moreover, immunoblotting demonstrated that LIMp27 knockdown caused upregulation of p27, but not p21 protein in p53‐mutant COAD cells (Figure 3a, Figure S4d, Supporting Information), pointing to the engagement of p27 by LIMp27 rather than p21. Indeed, co‐knockdown experiments showed that p27 but not p21 diminished the reductions in BrdU incorporation, clonogenicity, and G0/G1 cell cycle arrest triggered by LIMp27 knockdown in WiDr and HT‐29 cells (Figure 3b–e, Figure S4e–h, Supporting Information). Conversely, the overexpression of p27 abolished the increases in cell proliferation caused by LIMp27 overexpression (Figure S4i, Supporting Information). Of note, knockdown of LIMp27 also resulted in upregulation of p27 at the mRNA and protein levels in wild‐type HCT116 and RKO cells (Figure S4j, Supporting Information). Therefore, these results establish that inhibition of p27 mRNA expression plays an important role in LIMp27‐mediated promotion of cell proliferation in p53 mutant COAD.

We then evaluated how LIMp27 regulates p27 mRNA expression. Actinomycin D‐chase experiments showed that LIMp27 knockdown prolonged the half‐life of p27 mRNA in HT‐29 and WiDr cells (Figure 3f,g), suggesting that LIMp27 affected the stability of p27 mRNA rather than altering its transcription levels.^[^ ^11^, ^28^ ^]^ In support, knockdown of LIMp27 did not affect the enrichment of the transcriptional activation mark H3K4me3 and the transcriptional repression mark H3K27me3 at the CDKN1B promoter (Figure S4k,l, Supporting Information). Furthermore, overexpression of LIMp27 accelerated, albeit moderately, the turnover of p27 mRNA (Figure S4m,n, Supporting Information). Additional experiments using cycloheximide‐chase assays revealed that reducing LIMp27 expression in HT‐29 and WiDr cells did not alter the protein half‐life of p27 (Figure 3h,i), indicating that LIMp27 did not impinge on the turnover of the p27 protein. Together, these results demonstrate that LIMp27 represses the stability of p27 mRNA in COAD cells.

### 2.4 LIMp27 Interacts with Cytoplasmic hnRNPA0

**Figure 4:**
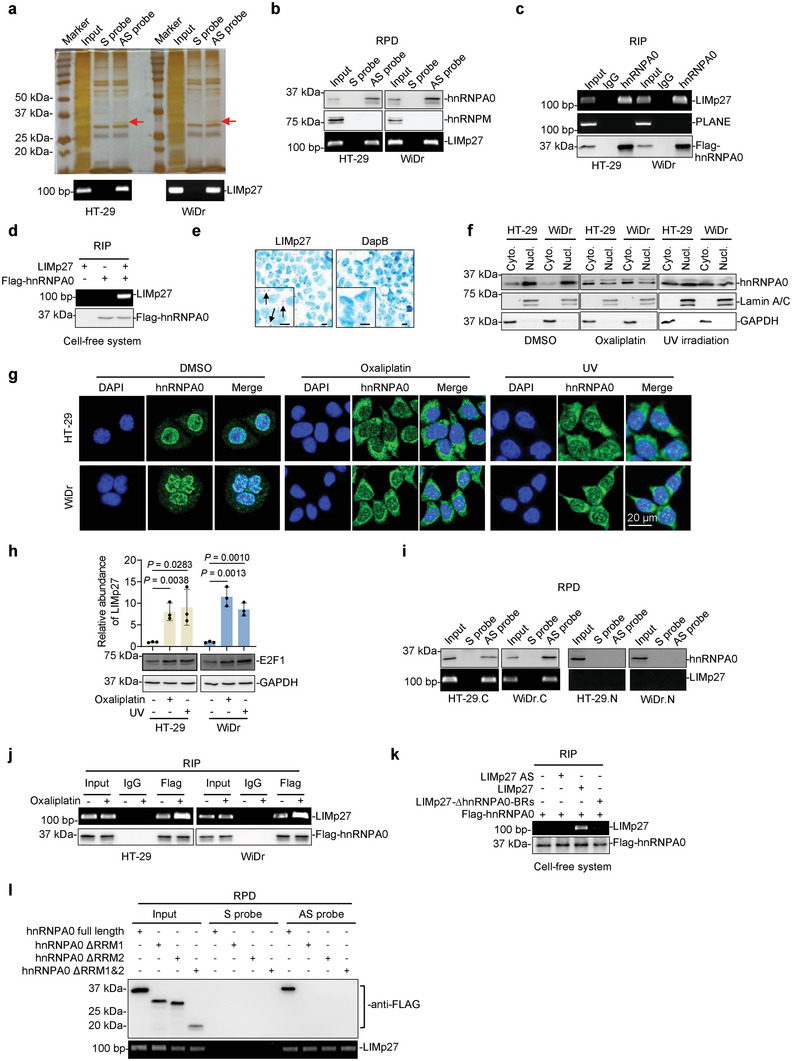
LIMp27 interacts with cytoplasmic hnRNPA0. a) RNA pulldown followed by mass spectrometry analysis identified that hnRNPA0 (indicated by arrows) is the most abundant protein co‐pulled down with LIMp27 antisense probes in HT‐29 and WiDr cell lines. S: sense; AS: antisense. *n* = 1 experiment. b) hnRNPA0 was co‐pulled down with LIMp27 in HT‐29 and Caco‐2 cell lines as shown in RNA pulldown (RPD) assays. hnRNPM was included as a negative control. S, sense; AS, antisense. Data are representatives of three independent experiments. c) LIMp27 was co‐precipitated with hnRNPA0 in HT‐29 and WiDr cell lines as shown in RNA immunoprecipitation (RIP) assays. The lncRNA PLANE was included as a negative control. Data are representatives of three independent experiments. d) In vitro‐synthesized LIMp27 was co‐precipitated with recombinant Flag‐tagged hnRNPA0 protein as shown in RIP assays in a cell‐free system. Data are representatives of three independent experiments. e) Representative microphotographs of in situ hybridization (ISH) analysis of LIMp27 expression in Caco‐2 cell line. DapB: negative control. Scale bar, 10 µm. Data are representatives of three independent experiments. f) Western blotting showing hnRNPA0 protein is mainly localized in the nucleus of HT‐29 and WiDr cell lines under steady‐state conditions. DNA damage inducers oxaliplatin (1 µm) and UV irradiation (10 J m^−2^) caused the relocation of hnRNPA0 from the nucleus to the cytoplasm. Lamin A/C and GAPDH were included as controls for nuclear and cytoplasmic fractions, individually. Data are representatives of three independent experiments, Cyto: cytoplasm; Nucl: nucleus. g) Immunofluorescence staining of hnRNPA0 in HT‐29 and WiDr cell lines treated with or without oxaliplatin (1 µm) or UV irradiation (10 J m^−2^). Scale bar, 20 µm. h) Oxaliplatin (1 µm) and UV irradiation (10 J m^−2^) caused upregulation of LIMp27 along with E2F1 expression in HT‐29 and WiDr cell lines. Data are representatives or mean ± s.d.; *n* = 3 independent experiments, One‐way ANOVA followed by Tukey's multiple comparisons test. i) hnRNPA0 was co‐pulled down with LIMp27 in cytoplasm but not nucleus in HT‐29 and WiDr cell lines. S, sense; AS, antisense; C: cytoplasm; N: nucleus. Data are representatives of three independent experiments. j) Oxaliplatin (1 µm) treatment increased the amount of LIMp27 associated with hnRNPA0 in HT‐29 and WiDr cell lines. Data are representatives of three independent experiments. k) In vitro‐synthesized LIMp27 but not LIMp27 antisense (AS) and LIMp27 with hnRNPA0‐BRs deletion, was co‐precipitated with recombinant Flag‐tagged hnRNPA0 protein in a cell‐free system as shown in RIP assays. Data are representatives of three independent experiments. l) full‐length hnRNPA0 protein but not hnRNPA0 with either RNA recognition motif 1 (RRM1) or RRM2 deletion mutant were co‐pulled down by LIMp27 as shown in RNA pulldown (RPD) assays. Data are representatives of three independent experiments.

To investigate the underlying mechanism whereby LIMp27 regulates p27 mRNA stability, we employed RNA‐pulldown (RPD) assays in combination with mass spectrometry to identify proteins that interact with LIMp27 in HT‐29 and WiDr cells (**Figure** **4**a). The most abundant protein that co‐precipitated with LIMp27 was hnRNPA0 (Figure S5a, Table S2, Supporting Information), with further analyses using RNA pulldown and RNA immunoprecipitation (RIP) assays confirming the association between LIMp27 and hnRNPA0 (Figure 4b,c). In contrast, there were no associations detected between LIMp27 and hnRNPM (Figure 4b) nor hnRNPA0 and the lncRNA PLANE (Figure 4c) that were included as controls in these respective experiments.^[^ ^26^ ^]^ Intriguingly, interactions between LIMp27 and hnRNPA0 were also detected in wild‐type p53 HCT116 and RKO cells (Figure S5b, Supporting Information). The physical association between LIMp27 and hnRNPA0 was due to their direct interaction, as in vitro‐synthesized LIMp27 coprecipitated recombinant hnRNPA0 in a cell‐free system (Figure 4d).

LIMp27 is primarily localized to the cytoplasm (Figure 4e, Figure S5c, Supporting Information), whereas hnRNPA0 is predominantly a nuclear protein that is nonetheless translocated to the cytoplasm in response to DNA damage in p53‐defective cancer cells.^[^ ^11^ ^]^ Given the tonic levels of DNA damage in cells, especially cancer cells,^[^ ^2^ ^]^ it is likely the observed binding of LIMp27 to hnRNPA0 is associated with the cytoplasmic pool of hnRNPA0 resulting from DNA damage under steady‐state conditions.^[^ ^11^, ^17^ ^]^ Indeed, while the majority of hnRNPA0 was found in the nucleus, a proportion of hnRNPA0 was clearly measurable in the cytoplasm in HT‐29 and WiDr cells (Figure 4f,g). As anticipated, exposure of cells to the DNA damage inducer oxaliplatin and UV irradiation resulted in marked relocation of hnRNPA0 from the nucleus to the cytoplasm (Figure 4f,g).^[^ ^29^, ^30^ ^]^ Moreover, consistent with the notion that DNA damage induces upregulation of E2F1,^[^ ^31^ ^]^ oxaliplatin and UV treatments led to increased E2F1 as well as LIMp27 expression (Figure 4h). Not surprisingly, the association between LIMp27 and hnRNPA0 was readily detected in the cytoplasmic fraction and was further enhanced after treatment with oxaliplatin (Figure 4i,j, Figure S5d, Supporting Information). Conversely, no interaction between LIMp27 and hnRNPA0 was found in the nuclear fraction from HT‐29 and WiDr cells (Figure 4i).

Comparable analyses of wild‐type p53 HCT116 and RKO cells also similarly revealed that a fraction of hnRNPA0 was present in the cytoplasm under steady‐state conditions and associated with LIMp27 (Figure S5e,f, Supporting Information). However, the cytoplasmic expression of hnRNPA0 was no longer measurable after treatment with oxaliplatin or UV irradiation, coinciding with the marked reduction in the overall levels of hnRNPA0 protein along with its mRNA (Figure S5e,g, Supporting Information). Nevertheless, oxaliplatin or UV irradiation treatment similarly caused upregulation of E2F1 and LIMp27 in these cells (Figure S5h, Supporting Information). These data support the notion that DNA damage destabilizes hnRNPA0 mRNA leading to downregulation of hnRNPA0 protein in wild‐type p53 cancer cells.^[^ ^11^ ^]^ Moreover, in agreement with the reported roles of p53/p21 in the downregulation of hnRNPA0 after DNA damage in wild‐type p53 cancer cells,^[^ ^11^ ^]^ knockdown of either p53 or p21 in HCT116 and RKO cells diminished the reductions in hnRNPA0 mRNA after oxaliplatin treatment or UV irradiation (Figure S5i,j, Supporting Information).

Notably, LIMp27 contains a consensus AU‐rich hnRNPA0 binding region (LIMp27‐hnRNPA0‐BR) located at nt 284–292 (Figure S6a, Supporting Information).^[^ ^32^ ^]^ Deletion of the LIMp27‐hnRNPA0‐BR abrogated its association with hnRNPA0 (Figure 4k), indicating this motif is responsible for the interaction of LIMp27 with hnRNPA0. Alternatively, the hnRNPA0 protein contains two RNA recognition motifs (RRMs) located at aa 7–86 and aa 98–175, respectively (Figure S6b, Supporting Information). Deletion of either RRM diminished associations between hnRNPA0 and LIMp27 (Figure 4l), demonstrating that both RRMs are required to support hnRNPA0‐LIMp27 interactions. Of note, neither knockdown nor overexpression of hnRNPA0 altered LIMp27 expression (Figure S6c,d, Supporting Information), and similarly, neither the knockdown nor overexpression of LIMp27 impinged upon the expression of hnRNPA0 (Figure S6e,f, Supporting Information). Thus, no regulatory relationships exist between hnRNPA0 and LIMp27.

### 2.5 LIMp27 Promotes p27 mRNA Degradation through Competitively Binding to Cytoplasmic hnRNPA0

**Figure 5:**
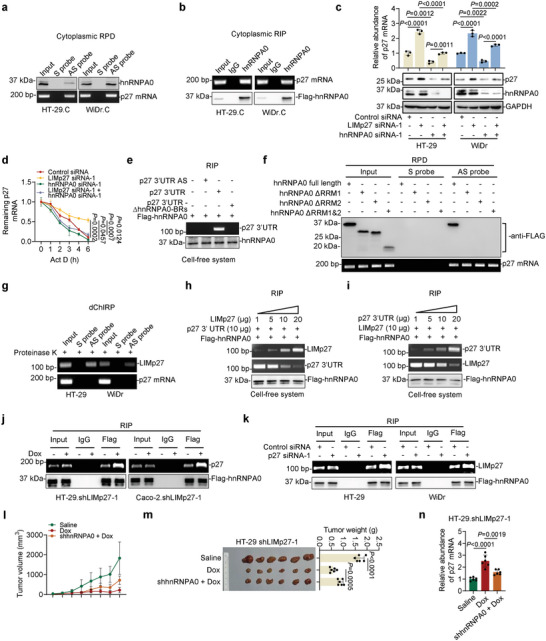
LIMp27 promotes p27 mRNA degradation through competitively binding to cytoplasmic hnRNPA0. a) Cytoplasmic hnRNPA0 was co‐pulled down with p27 mRNA in HT‐29 and WiDr cell lines as shown in RNA pulldown (RPD) assays. C: cytoplasm. Data are representatives of three independent experiments. b) p27 mRNA was co‐precipitated with hnRNPA0 in the cytoplasmic faction of HT‐29 and WiDr cell lines as shown in RNA immunoprecipitation (RIP) assays. C: cytoplasm. Data are representatives of three independent experiments. c,d) The upregulation of p27 expression (c) and increased stability of p27 mRNA (d) upon LIMp27 silencing ware reversed by knockdown of hnRNPA0. Data are representatives or mean ± s.d.; *n* = 3 independent experiments, One‐way ANOVA followed by Tukey's multiple comparisons test. e) In vitro‐synthesized p27 3′UTR was co‐precipitated with recombinant Flag‐tagged hnRNPA0 protein as shown in RIP assays in a cell‐free system. Data are representatives of three independent experiments. f) full‐length hnRNPA0 protein but not hnRNPA0 with either RNA recognition motif 1 (RRM1) or RRM2 deletion mutant was co‐pulled down by p27 mRNA as shown in RNA pulldown assays. Data are representatives of three independent experiments. g) LIMp27 was not co‐precipitated with the endogenous p27 mRNA as shown in dChIRP assays. Probes against LIMp27 RNA were used. Data are representatives of three independent experiments. h) Increasing amounts of in vitro‐synthesized LIMp27 (1, 5, 10, 20 µg) incubated with certain amounts of the 3′UTR of the p27 mRNA (10 µg) and recombinant hnRNPA0 were subjected to RIP assay. Data are representatives of three independent experiments. i) Increasing amounts of in vitro‐synthesized 3′UTR of p27 mRNA (1, 5, 10, 20 µg) incubated with certain amounts of the LIMp27 (10 µg) and recombinant hnRNPA0 were subjected to RIP assay. Data are representatives of three independent experiments. j) Induced knockdown of LIMp27 increased the amount of hnRNPA0 associated with p27 mRNA. Data are representatives; n = 3 independent experiments. k) Knockdown of p27 increased the amount of hnRNPA0 associated with LIMp27. Data are representatives; *n* = 3 independent experiments. l–n) Growth curves (l) and representative photographs and tumor weights (m) showing induced knockdown of LIMp27 by Dox increased p27 mRNA expression (n) and retarded HT‐29.shLIMp27 xenograft growth, which was reversed by hnRNPA0 stable knockdown in nu/nu mice. Data are mean ± s.d.; *n* = 6 mice per group, one‐way ANOVA followed by Tukey's multiple comparison test. Dox: 2 mg mL^−1^ supplemented with 10 mg mL^−1^ sucrose in drinking water.

Cytoplasmic hnRNPA0 is known to bind and stabilize p27 mRNA.^[^ ^11^ ^]^ Indeed, hnRNPA0 and p27 mRNA were co‐precipitated from cytoplasmic fractions of HT‐29 and WiDr cells (**Figure** **5**a,b). Moreover, knockdown of hnRNPA0 caused reductions in p27 mRNA levels (Figure S7a, Supporting Information), whereas co‐knockdown of hnRNPA0 diminished the regulatory effect of LIMp27 knockdown on p27 mRNA expression and p27 mRNA half‐life (Figure 5c,d), indicating that hnRNPA0 is necessary for LIMp27‐mediated destabilization of p27 mRNA.

The p27 mRNA also contains two putative AU‐rich hnRNPA0 binding regions (p27 mRNA‐hnRNPA0‐BRs) at its 3′UTR (Figure S7b, Supporting Information).^[^ ^11^, ^32^ ^]^ In accordance, hnRNAPA0 co‐precipitated an RNA fragment containing this region in a cell‐free system, but this association was abolished when the p27 mRNA‐hnRNPA0‐BRs were deleted (Figure 5e), supporting the notion that the p27 mRNA 3′UTR is responsible for interacting with hnRANPA0.^[^ ^11^ ^]^ Furthermore, drawing parallels with the binding of hnRNPA0 to LIMp27 (Figure 4l), the deletion of either of the two RRMs in hnRNPA0 diminished interactions between hnRNPA0 and p27 mRNA (Figure 5f). Thus, both RRMs are necessary for hnRNPA0 associations with LIMp27 and p27 mRNA. Nevertheless, no direct interactions were detected between LIMp27 and p27 mRNA as shown using domain‐specific chromatin isolation by RNA purification (dChIRP) assays (Figure 5g), indicating that hnRNPA0, LIMp27 and p27 mRNA do not form a ternary structure.

We next considered the alternative hypothesis that LIMp27 and p27 mRNA compete for interactions with hnRNPA0. This notion was tested in a cell‐free system adding in vitro‐synthesized LIMp27 to a fixed amount of p27 mRNA‐hnRNPA0‐BR RNA fragments and recombinant hnRNPA0. Notably, adding increasing amounts of LIMp27 caused progressive reduction in the association between the p27 RNA fragment and hnRNPA0 (Figure 5h). Importantly, deletion of the hnRNPA0‐BR in LIMp27 abolished this effect (Figure S7c, Supporting Information), suggesting that LIMp27‐hnRNAPA0 binding interactions can attenuate binding between p27 mRNA and hnRNPA0. An alternative approach adding increasing amounts of the p27 mRNA‐hnRNPA0‐BR‐containing fragment to a fixed mixture of LIMp27 and hnRNPA0 similarly resulted in gradual decreases in LIMp27 and hnRNPA0 interactions (Figure 5i). And consistent with prior experiments, deletion of the p27 mRNA‐hnRNPA0‐BR abolished this effect (Figure S7d, Supporting Information). Together these data confirmed that LIMp27 and p27 mRNA compete for binding to hnRNPA0 in an in vitro context.

Consistent with the findings of these assays, knockdown of LIMp27 in HT‐29 and WiDr cells caused increases in the amounts of p27 mRNA associated with hnRNPA0, whereas overexpression of LIMp27 led to decreases in their association (Figure 5j, Figure S7e, Supporting Information). Similarly, knockdown of p27 resulted in an increase in the amount of LIMp27 binding to hnRNPA0, whereas overexpression of p27 reduced their binding (Figure 5k, Figure S7f, Supporting Information). Importantly, these findings were phenocopied in nu/nu mouse experiments whereby the stable knockdown of hnRNPA0 in HT‐29.shLIMp27 xenografts rescued the effects of LIMp27 knockdown, reversing decreases in tumor growth and increases in the expression of p27 mRNA (Figure 5l–n). The introduction of exogenous wild‐type LIMp27 but not the LIMp27‐hnRNPA0‐BR‐deleted mutant into HT‐29.shLIMp27 and Caco‐2.shLIMp27 cells reversed the increase in p27 expression and inhibition of cell proliferation caused by knockdown of endogenous LIMp27 (Figure S7g,h, Supporting Information), indicating the hnRNPA0‐BR is essential for the effect of LIMp27 on cell viability. Further consolidating this, CRISPR/Cas‐mediated disruption of the hnRNPA0‐BR at the LIMp27 genomic locus diminished the binding between LIMp27 and hnRNPA0 (Figure S7i–k, Supporting Information). Instructively, LIMp27 siRNA knockdown failed to alter p27 expression and cell viability in cells with the hnRNPA0‐BR knocked out (Figure S7l,m, Supporting Information). Collectively, these results demonstrated that LIMp27 competes with p27 mRNA for binding to hnRNPA0, thus destabilizing p27 mRNA.

### 2.6 LIMp27 Regulates Mutant p53 COAD Cell Responses to DNA‐Damaging Therapeutics

**Figure 6:**
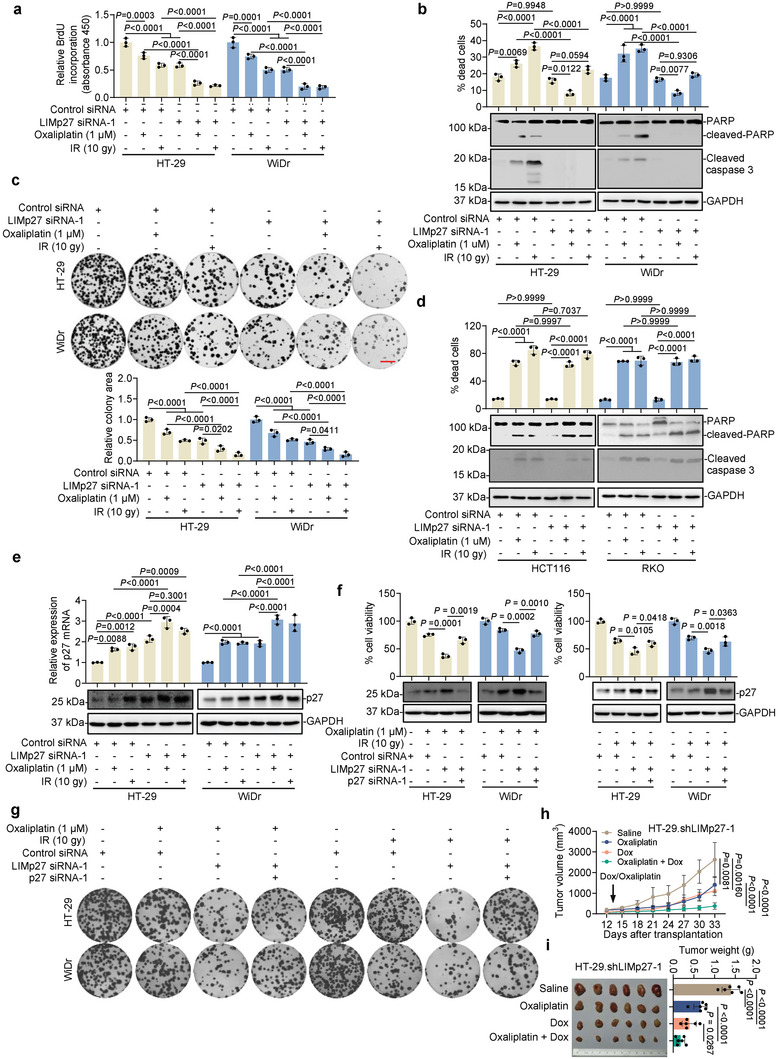
LIMp27 regulates mutant p53 COAD cell responses to DNA‐damaging therapeutics.  a–c) HT‐29 and WiDr cells transfected with LIMp27 siRNA and treated with or without oxaliplatin (1 µm) for 24 h or IR (10 gy) were subjected to BrdU incorporation (a), cell death assay and Western blot (b) and clonogenicity assay (c). Data are mean ± s.d. or representatives; *n* = 3 independent experiments, One‐way ANOVA followed by Tukey's multiple comparisons test. Scale bar, 1 cm. d) HCT116 and RKO cells transfected with LIMp27 siRNA and treated with or without oxaliplatin (1 µm) for 24 h or IR (10 gy) were subjected to cell death assay and Western blot. Data are mean ± s.d.; *n* = 3 independent experiments, One‐way ANOVA followed by Tukey's multiple comparisons test. e) HT‐29 and WiDr cells transfected with LIMp27 siRNA and treated with or without oxaliplatin (1 µm) for 24 h or IR (10 gy) were subjected to qPCR and Western blot. Data are mean ± s.d.; *n* = 3 independent experiments, One‐way ANOVA followed by Tukey's multiple comparisons test. f,g) LIMp27 knockdown further inhibited cell viability (f) and clonogenicity (g), which was reversed by co‐knockdown of p27 in HT‐29 and WiDr cells treated with oxaliplatin (1 µm, left) and IR (10 gy, right). Data are mean ± s.d.; *n* = 3 independent experiments, One‐way ANOVA followed by Tukey's multiple comparisons test. h,i) Growth curves (h) and representative photographs and tumor weights (i) showing HT‐29.shLIMp27 xenografts in nu/nu mice treated with or without Dox (1 mg mL^−1^ supplemented with 10 mg mL^−1^ sucrose in drinking water) and/or oxaliplatin (5 mg kg^−1^ by intraperitoneal injection, twice a week). Data are representatives or mean ± s.d.; *n* = 6 mice per group, one‐way ANOVA followed by Tukey's multiple comparison test.

P27 is critical for the establishment of the G1/S checkpoint in response to DNA damage in p53‐defective cells.^[^ ^11^ ^]^ whereas our data now establish that LIMp27 plays an important role in p27 regulation (Figure 3a).^[^ ^11^ ^]^ Based on these results, we tested whether LIMp27 was involved in regulating cell responses to DNA‐damaging therapeutics in the context of COAD. The combination of LIMp27 knockdown with oxaliplatin or ionizing radiation (IR) cooperatively inhibited HT‐29 and WiDr cell proliferation (**Figure** **6**a). Paradoxically, LIMp27 knockdown rendered HT‐29 and WiDr cells more resistant to apoptosis after oxaliplatin and IR, although the proportion of apoptotic cells resulting from oxaliplatin or IR treatment was low in HT‐29 and WiDr cells even without LIMp27 knockdown (Figure 6b). Regardless, LIMp27 knockdown and oxaliplatin or IR cooperatively reduced cell clonogenicity (Figure 6c), demonstrating that LIMp27 predominantly functions in mutant p53 COAD cells to protect against oxaliplatin‐ and IR‐induced proliferation inhibition. In support, the overexpression of LIMp27 in HT‐29 and WiDr cells blunted the inhibition of cell proliferation and clonogenicity caused by oxaliplatin and IR although it did not significantly impinge on apoptosis (Figure S8a–c, Supporting Information). The cooperative inhibitory effect of LIMp27 knockdown and oxaliplatin or IR treatment on cell proliferation was also observed in additional p53 mutant COAD cell lines (Figure S8d, Supporting Information). Furthermore, LIMp27 knockdown enhanced oxaliplatin‐mediated inhibition of p53 mutant MDA‐MB‐231 and H226 cell proliferation and rendered cells more resistant to oxaliplatin‐induced apoptosis (Figure S8e,f, Supporting Information). Conversely, however, treating wild‐type p53 HCT116 and RKO cells with oxaliplatin or IR induced marked apoptotic cell death irrespective of LIMp27 expression (Figure 6d).^[^ ^33^ ^]^

We further sought to corroborate the role of p27 in LIMp27‐mediated protection against DNA‐damaging therapeutics. Exposure to oxaliplatin or IR increased p27 expression in HT‐29 and WiDr cells (Figure 6e), with more marked upregulation when LIMp27 was knocked down (Figure 6e). Furthermore, the knockdown of p27 attenuated the cooperativity between LIMp27 knockdown and oxaliplatin or IR treatment in inhibiting HT‐29 and WiDr cell viability (Figure 6f), whereas ectopic p27 expression diminished the inhibitory effects of LIMp27 overexpression on cell proliferation after oxaliplatin or IR‐treatments (Figure S8g, Supporting Information). Thus, inhibition of p27 is responsible for LIMp27‐mediated protection of p53‐mutant COAD cells against DNA‐damaging therapeutics.

Last, to substantiate the significance of LIMp27 with respect to in vivo tumor responses against DNA‐damaging drugs, we treated nu/nu mice carrying HT‐29.shLIMp27 and HT‐29.shCtrl xenografts with Dox, oxaliplatin, or Dox in combination with oxaliplatin. Instructively, co‐treatment of mice with Dox and oxaliplatin resulted in markedly greater inhibition of tumor growth of HT‐29.shLIMp27 but not HT‐29.shCtrl in comparison with Dox or oxaliplatin treatments alone (Figure 6h,i, Figure S8h, Supporting Information). The reduced tumor burden was associated with decreases in LIMp27 and increases in p27 expression and decreased proportions of Ki67 positive cells (Figure S8i–k, Supporting Information). These data confirmed that LIMp27 plays an important role in governing the response of p53‐defective COADs to DNA damage in vivo.

## 3 Discussion

The tumor suppressor p53 regulates the DNA damage response through its “gatekeeper” function, arresting cell cycle progression to allow sufficient time for DNA repair, or, upon irreversible DNA damage, eliminating cells through apoptosis.^[^ ^4^ ^]^ We have previously shown that the lncRNA GUARDIN acts pleiotropically downstream of p53 to maintain genomic integrity in cells under steady‐state conditions and after exposure to exogenous genotoxic stress, suggestive that targeting GUARDIN represents a potential treatment approach for targeting cancers carrying wild‐type p53.^[^ ^1^ ^]^ However, this approach is inherently limited given that p53 is lost or mutated in approximately half of human cancers, also being closely associated with treatment resistance and poor patient outcomes.^[^ ^4^, ^34^ ^]^ This study now demonstrates a hitherto unrecognized mechanism involving the lncRNA LIMp27 in the mutant p53 setting. Driven by E2F1 transactivation, not only does LIMp27 promote cell proliferation, tumorigenicity and resistance to DNA‐damaging therapeutics, but a selective connection was made between LIMp27 and the regulation of the DDR in p53‐defective COAD cells. Together this highlights the potential utility of LIMp27 as a treatment target for p53‐defective cancers but also suggests that LIMp27 targeting could be used to counteract the cancer‐promoting axis of E2F1 signaling.^[^ ^35^ ^]^

LIMp27 promoted p53‐mutant COAD cell proliferation and tumorigenicity through inhibition of the expression of p27 with reduced stability of p27 mRNA identified as the primary mechanism. As a CDK inhibitor, p27 plays a role in controlling the G1/S checkpoint^[^ ^15^ ^]^ and becomes essential for the establishment of the G1/S checkpoint in p53‐defective cells where p53‐driven p21 activity is absent.^[^ ^11^ ^]^ Working off the prior elegant demonstration that DNA damage in p53‐defective cancer cells results in the stabilization of p27 mRNA through binding to cytoplasmic hnRNPA0,^[^ ^11^ ^]^ we observed that the proportion of hnRNPA0 in the cytoplasm of COAD cells markedly increased upon exogenous genotoxic stress as a result of nuclear translocation.^[^ ^11^, ^36^ ^]^ LIMp27 was predominantly located to the cytoplasm and was upregulated after induction of DNA damage with clear indications that a competitive relationship exists between LIMp27 and p27 mRNA in binding to cytoplasmic hnRNPA0. The supporting evidence included: 1) LIMp27, similar to p27 mRNA, was physically associated with hnRNPA0 in the cytoplasm; 2) LIMp27 and p27 mRNA bound to the same RRMs on hnRNPA0; 3) LIMp27 and p27 mRNA did not directly interact with each other; 4), LIMp27 and p27 competed with each other for binding to hnRNPA0 in a cell‐free system, which was however interrupted when the LIMp27‐hnRNPA0‐BR or the p27 mRNA‐hnRNPA0‐BR contained in LIMp27 and p27 mRNA, respectively, was deleted; and 5), knockdown of endogenous LIMp27 led to an increased association between p27 mRNA and hnRNPA0, whereas knockdown of endogenous p27 promoted the interaction between LIMp27 and hnRNPA0. As a result, competition of LIMp27 with p27 mRNA for binding to cytoplasmic hnRNPA0 regulates p27 expression and consequently, exerts control over the G1/S checkpoint in p53‐mutant COAD cells. Nevertheless, it is intriguing to consider the relative abundance of LIMp27 with respect to that of hnRNPA0.

Like other hnRNP family proteins, hnRNPA0 is highly abundant in cells with a previous quantitative proteomic study showing that it was present with 232 000 molecules per U2OS cell.^[^ ^37^ ^]^ In comparison, LIMp27 expression is of markedly lower abundance that we estimated at ≈ 124 molecules per HT‐29 cell. However, there are several physiological aspects that may help explain how the competition model functions in this setting. First, interaction between LIMp27 and hnRNPA0 occurred in the cytoplasm, where only a fraction of hnRNPA0 was located under steady‐state conditions. Moreover, when cytoplasmic hnRNPA0 was increased upon DNA damage, the abundance of LIMp27 was also upregulated. Furthermore, from the perspective of p27 mRNA, altering its half‐life would be expected to amplify changes in the overall levels of translated p27 protein. Whether these molecules also exist and interact within a specialized cytoplasmic subdomain is also not clear. But considering such factors it is conceivable that an adequate stoichiometric basis exists to explain how LIMp27 competes with p27 mRNA for interaction with hnRNPA0. Nevertheless, some aspects of this model require further investigation.

In contrast to its effect in p53‐mutant cells, LIMp27 did not appear to play any proliferative role associated with wild‐type p53. But then what determines such specificity toward p53‐mutant COAD cells? Since LIMp27 expression is comparable in mutant and wild‐type p53‐bearing cells both before and after the induction of DNA damage, this argues against expression differences being responsible. Moreover, in wild‐type p53 COAD cells, LIMp27 bound to cytoplasmic hnRNPA0 and knockdown of LIMp27 caused p27 upregulation under steady‐state conditions, demonstrating that the competitive ability of LIMp27 to regulate p27 expression through hnRNPA0 binding remains intact. Noticeably, wild‐type p53 establishes the G1/S checkpoint primarily through transcriptionally activating p21,^[^ ^5^, ^38^ ^]^ whereas p27 becomes essential for maintaining the control only when the initial wave of p53‐driven p21 activity is diminished.^[^ ^11^ ^]^ As such, a biological model has been proposed where p27 controls the G1/S checkpoint in cancer cells lacking an active p53/p21 pathway.^[^ ^11^, ^39^ ^]^ Therefore, in wild‐type p53 CRC cells under steady‐state conditions, p53/p21 signaling overrides p27 signaling even when p27 is upregulated in cells with LIMp27 knocked down. Furthermore, as demonstrated previously and substantiated by our results,^[^ ^11^ ^]^ p53/p21 signaling destabilized hnRNPA0 mRNA, downregulating its expression and diminishing its presence in the cytoplasm in wild‐type p53 cancer cells upon induction of DNA damage. This prevents LIMp27 from destabilizing the p27 mRNA through hnRNPA0 in the cytoplasm. Thus, the p53‐driven p21 activity is the major determinant of the inability of LIMp27 to regulate proliferation in wild‐type p53 COAD cells (Figure S5i,j, Supporting Information). Whether LIMp27 expressed in wild‐type p53 cells has biological functions other than regulating p27 mRNA stability remains unknown, but it is likely to function as a backup mechanism to control the G1/S checkpoint in case the p53/p21 machinery becomes impaired.

As a transcription factor, E2F1 is upregulated in response to DNA damage and can exert dual but contrasting functions in a cell type‐ and context‐dependent manner.^[^ ^40^ ^]^ On the one hand, it transactivates many protein‐coding genes involved in cell cycle progression and its high expression is associated with tumorigenesis.^[^ ^41^ ^]^ On the other hand, E2F1 can mediate apoptotic cell death and its loss has been demonstrated to induce cancer development and progression.^[^ ^40^, ^42^ ^]^ Specifically, E2F1 transcriptionally activates p27, which has been suggested to be a negative feedback mechanism for E2F1 promotion of cell cycle progression.^[^ ^43^ ^]^ Our results identified transcriptional activation of LIMp27 as a mechanism that negatively regulates p27 expression downstream of E2F1, suggesting that E2F possesses dichotomous functions in controlling p27 expression. This safeguard provides fine‐tuning of the G1/S checkpoint, in particular, in p53‐defective cells where p53/p21 mechanisms are disabled. Thus, LIMp27 may represent a potential target for counteracting the cancer‐promoting axis of E2F1 signaling in the p53‐defective cancer.^[^ ^44^ ^]^

A practical implication of this study is the potential application in the treatment of p53‐mutant CRC, and likely many other types of cancer with defects in p53. Knockdown of LIMp27 not only inhibited p53‐mutant CRC cell proliferation and tumorigenicity, but also cooperated with genotoxic drugs to inhibit p53‐mutant CRC xenograft growth. Of particular interest, a number of first‐line chemotherapeutic drugs in CRC treatment, such as 5‐fluorouracil (5‐FU) and oxaliplatin, exert their therapeutic effects through causing damage to DNA.^[^ ^45^, ^46^ ^]^ Targeting LIMp27 using Gapmer technology, nanoparticles loaded with LIMp27 siRNA, small molecule compounds, or small molecules that block the interaction of LIMp27 with hnRNPA0 all represent potential investigative avenues to advance our findings toward clinical applications.^[^ ^47^, ^48^, ^49^ ^]^

Last, our study sheds light on the paradox whereby hnRNPA0‐mediated stabilization of p27 mRNA serves to protect cells against killing by cytotoxic drugs, particularly conferring chemoresistance in p53‐defective tumors.^[^ ^11^, ^34^, ^50^ ^]^ This implies that LIMp27‐mediated downregulation of p27 would sensitize these tumors to genotoxic chemotherapeutics, whereas LIMp27 targeting would confer resistance. Nevertheless, our results showed that p53‐mutant COAD cells are largely resistant to cell death induced by DNA damage. This is presumably due to the lack of the p53‐driven expression of pro‐apoptotic proteins such as PUMA and NOXA.^[^ ^4^, ^38^ ^]^ Therefore, the therapeutic effect of DNA‐damaging treatment in p53‐defective CRCs is primarily achieved through the inhibition of cell proliferation. Moreover, our results have clearly demonstrated that the biological function of LIMp27 in p53‐defective COAD cells is mainly to promote proliferation through down‐regulating p27. Thus, LIMp27 targeting, alone and in combination with other DNA‐damage inducers, will be beneficial to patients with p53‐defective CRCs. Indeed, high LIMp27 expression is associated with poor OS of such patients, whereas high p27 expression is also an indicator of unfavorable prognosis of patients with p53‐defective CRCs.^[^ ^12^ ^]^

## 4 Experimental Section

### Cell Culture and Human Tissue

The human normal colon epithelial cell line FHC (ATCC CRL‐1831), the human COAD cell lines HT‐29 (ATCC HTB‐38), WiDr (ATCC CCL‐218), Caco‐2 (ATCC HTB‐37), HCT116 (ATCC CCL‐247), RKO (ATCC CRL‐2577), SW480 (ATCC CCL‐228), human breast cancer cell lines MCF‐7 (ATCC HTB‐22), MDA‐MB‐231 (ATCC HTB‐26), human non‐small cell lung cancer cell lines A549 (ATCC CCL‐185), H226 (ATCC CRL‐5826), and human embryonic kidney cell line HEK293T were from American Type Culture Collection (ATCC). The human COAD cell line LIM1215 (ECACC 10 092 301) was from the European Collection of Authenticated Cell Cultures (ECACC). Cells were cultured according to standard mammalian tissue culture protocols. All cell lines were verified to be free of mycoplasma contamination every 3 months and were authenticated by short tandem repeat (STR) profiling by the Australian Genome Research Facility (AGRF). Studies using FFPE tissue arrays (HColA160CS01), including paired normal colon mucosa and COAD tissues, were purchased from Shanghai Outdo Biotech Co., Ltd. Studies using human tissues were approved by the human ethics Review Committee of Shanghai Outdo Biotech Co., Ltd. (SHYJS‐CP‐1701008/YB M‐05‐02) in agreement with the guidelines set forth by the Declaration of Helsinki. The study is compliant with all relevant ethical regulations for human research.

### Antibodies and Reagents

Information on antibodies and reagents used in this study is provided in Tables S3 and S4, Supporting Information.

### Small interference RNA and short hairpin RNA

siRNAs were obtained from GenePharma (Shanghai, China) and transfected using Lipofectamine 3000 reagent (Invitrogen). ShRNA sequences were constructed into FH1‐tUTG inducible knockdown vector (Kind gift from Prof Herold MJ, WEHI). The lentiviral particles were packaged via co‐transfection with FH1‐tUTG, pMDLg.pRRE, pMD2.g, and pRSU.pREV plasmids into HEK293T cells. HT‐29 or Caco‐2 inducible knockdown cell sub‐lines were established after the lentiviral transduction. siRNA and shRNA sequences are shown in Table S5, Supporting Information.

### Plasmids

The FH1‐tUTG plasmid was a kind gift from Prof M. J. Herold (Walter and Eliza Hall Institute of Medical Research, Australia). The pMDLg/pRRE plasmid (#12 251), pMD2.g plasmid (#12 259), pRSU.pREV plasmid (#12 253), p27 cDNA (#20 420) and pCMVHA E2F1 (#24 225) were purchased from Addgene. The pGL3‐*LIM27*‐promoter and the pGL3‐LIMp27‐promoter‐ΔE2F1‐BR were constructed by Azenta Life Sciences (Suzhou, China). Other plasmids used in this study were generated by inserting the PCR products into the pcDNA3.1(+) or pcDNA3.1‐Flag vector by Azenta Life Sciences (Suzhou, China).

### Quantitative PCR

Total cellular RNA isolated using an ISOLATE II RNA Mini Kit (Bioline) was subjected to PCR analysis. The 2−ΔΔCT method was used to calculate the relative gene expression levels in comparison to the RPL13A housekeeping controls. Primer sequences are listed in Table S6, Supporting Information.

### Absolute quantification of LIMp27

Absolute RNA quantification was achieved using a standard curve constructed by amplifying known amounts of pcDNA3.1‐LIMp27 plasmids.^[^ ^25^ ^]^ cDNA was prepared from a certain number of cells using the qScript cDNA SuperMix (Quantabio, Cat#95048‐500) in a 20 µL reaction and subsequently diluted to 100 µL. Tenfold serial dilutions of pcDNA3.1‐LIMp27 plasmid were used to construct standard curves. Assays were reconstituted to a final volume of 20 µL using 5 µL cDNA/standard and amplified using a QuantStudio 6 Pro Real‐Time PCR System. Data calculated as copies per 5 µL cDNA were converted to copies per cell based on the known input cell equivalents. Primer sequences used are listed in Table S6, Supporting Information.

### CRISPR/Cas9‐Mediated hnRNPA0‐BR Deletion

Single‐guide (sg) RNAs targeting the hnRNPA0‐BR at LIMp27 genomic locus were constructed into the BsmBI‐digested lentiCRISPR v2 vector (Addgene plasmid #52 961) before transfection and selection using puromycin (2 µg mL^−1^) (Figure S7i, Supporting Information). Genomic DNA was extracted from single cell clones using the Wizard SV Genomic DNA Purification System (Promega, Cat#A2361) and genomic DNA flanking the CRISPR‐targeted region was amplified by PCR using the AmpliTaq Gold 360 Master Mix (Applied Biosystems, Cat#4 398 881). PCR products were purified using the Isolate II PCR and Gel Kit (Bioline, Cat#BIO‐52060) and analyzed by Sanger sequencing (Figure S7j, Supporting Information). The hnRNPA0‐BR sgRNA sequences and PCR primers used are listed in Tables S5 and S6, Supporting Information, respectively.

### Subcellular Fractionation

Cells were incubated with hypotonic buffer A (10 mm Hepes pH 7.9, 10 mm KCl, 0.1 mm EDTA, 0.1 mm EGTA, 1 mm DTT, 0.15% Triton X‐100, cOmplete, EDTA‐free Protease Inhibitor Cocktail) and ice for 10 min. Samples were centrifuged at 1000 × g for 10 min, and the supernatant was collected as the cytoplasmic fraction. The pellets were rinsed twice with cold hypotonic buffer A and nuclear proteins were extracted using an equal volume of buffer B (20 mm Hepes pH 7.9, 400 mm NaCl, 1 mm EDTA, 1 mm EGTA, 1 mm DTT, 0.5% Triton X‐100, cOmplete, EDTA‐free Protease Inhibitor Cocktail) on ice for 15 min. Cytoplasmic and nuclear fractions were subjected to qPCR or Western blotting analysis.

### In Situ Hybridization

ISH assays were performed using the BaseScope Reagent Kit v2‐Red (Advanced Cell Diagnostics, Hayward, CA) according to the manufacturer's instructions. Briefly, FFPE tissue sections (4 µm thick) were deparaffinized and rehydrated, then heated and treated with proteinase K. Sections were then hybridized with probes at 40 °C for 3 h. After washing, the sections were incubated with BaseScope Fast RED, and counterstaining was carried out using hematoxylin. Positive staining was identified as red, punctate dots present in cells. Reactive score (RS) was derived by determining i) the percentage of positive (LIMp27 stained) cells from 0 to 100% and ii) the staining intensity (intensity score) judged on an arbitrary scale of 0–4, that is, no staining (0), weakly positive staining,^[^ ^1^ ^]^ moderately positive staining,^[^ ^2^ ^]^ strongly positive staining,^[^ ^3^ ^]^ and very strong positive staining.^[^ ^4^ ^]^ RS was then calculated by multiplying the percentage by the staining intensity and dividing by 10.

### Biotin RNA Pulldown

Cell lysates were prepared by ultrasonication in lysis buffer (50 mm Tris‐HCl [pH 7.5], 150 mm NaCl, 2.5 mm MgCl_2_, 1 mm EDTA, 10% Glycerol, 0.5% Nonidet P‐40/Igepal CA‐630, 1 mm DTT, cOmplete EDTAfree Protease Inhibitor Cocktail and RNase inhibitors). Biotin‐labeled probes were incubated with lysate at 4 °C overnight before rotating with Streptavidin Agarose (ThermoFisher Scientific, SA10004) for an additional 3 h. Then beads were washed in lysis buffer five times and the binding complexes were eluted for further analysis. Probe sequences are shown in Table S7, Supporting Information.

### Mass Spectrometry Analysis

Proteins co‐pulled down with RNA using antisense/sense biotin‐labeled probes were separated by 10% acrylamide gels and visualized by silver staining. The specific protein band shown in the group using antisense probes along with the corresponding region in the group using sense probes were resected followed by in‐gel enzymatic digestion and extraction of peptides for subsequent analysis by mass spectrometry. Peptides were analyzed using a nanoflow liquid chromatography instrument (Thermo Dionex, Ultimate 3000 RSLCnano; Thermo Scientific, Waltham, MA, USA) connected to a Q‐Exactive Plus Orbitrap mass spectrometer (Thermo Scientific, Waltham, MA, USA). Proteins identified from the mass spectrometry analysis are listed in Table S2, Supporting Information. The raw mass spectrometry data were deposited in the public dataset MassIVE using the access number MSV000090292.

### RNA Immunoprecipitation

RIP was performed with an EZ‐Magna RIP Kit (17‐701; Millipore) according to the manufacturer's instructions. Briefly, 2 × 10^7^ cells were lysed in hypotonic buffer supplemented with RNase inhibitor and protease inhibitor before centrifugation. Cell lysates were incubated with magnetic beads coated with the indicated antibodies at 4 °C overnight. After extensive washing using RIP wash buffer, the bead‐bound immunocomplexes were eluted by lysis buffer and subjected to Western blotting analysis. To isolate RNAs, samples were centrifuged and placed on a magnetic separator, and supernatants were used to extract RNA by an ISOLATE II RNA Mini Kit (Bioline). Purified RNAs were then subjected to PCR analysis.

### Chromatin Immunoprecipitation

The ChIP assays were performed by using the MAGnify Chromatin Immunoprecipitation System (Thermo Fisher, 492 024) according to the manufacturer's instructions. The bound DNA fragments were subjected to PCR using specific primers. Primers used in this study are shown in Table S6, Supporting Information.

### In Vitro Transcription

The pcDNA3.1‐LIMp27 plasmids were linearized by restriction enzyme NotI (New England biolab) and in vitro transcription was then performed using TranscriptAid T7 High Yield Transcription Kit (ThermoFisher Scientific, K0441) according to the manufacturer's instructions.

### Flag‐hnRNPA0 Protein Purification

Flag‐hnRNPA0 protein was purified with the Pierce Anti‐DYKDDDDK Magnetic Agarose (A36797; Thermo Fisher Scientific) according to the manufacturer's instructions. Briefly, the pcDNA3.1‐Flag‐hnRNPA0 plasmids were transfected into HEK293T cells for 48 h and then cell pellets were collected and lysed using lysis buffer (25 mm Tris•HCl pH 7.4, 150 mm NaCl, 1% NP‐40, 1 mm EDTA, 5% glycerol). The lysates were then incubated with rotation in the presence of Pierce DYKDDDDK Magnetic Agarose at room temperature for 2 h. After extensive washing using PBS, the Flag‐hnRNPA0 proteins were eluted by Pierce 3× DYKDDDDK Peptide (A36805; Thermo Fisher Scientific) in PBS.

### Luciferase reporter assays

Assays were performed according to the manufacturer's instructions (Promega). Cells were transfected with the pGL3‐based constructs containing LIMp27 promoter together with Renilla luciferase plasmids. 24 h later, firefly and Renilla luciferase activities were examined by Dual‐Luciferase Reporter Assay System (Promega) and Renilla luciferase activities were used to normalize the firefly luciferase activity.

### Domain‐Specific Chromatin Isolation by RNA Purification

dChIRP assays were performed as previously described.^[^ ^26^ ^]^ Briefly, HT‐29 and WiDr cells were harvested and cross‐linked in 1% glutaraldehyde for 10 min at room temperature (RT) with rotation. The cross‐linked cells were lysed in lysis buffer (50 mm Tris‐Cl [pH 7.0], 10 mm EDTA, 1% SDS, PMSF, SUPERase‐in RNase inhibitor), followed by sonication. Three micrograms antisense/sense biotin‐labeled probes against LIMp27 RNA were rotated with cell lysates at 37 °C for 4 h, followed by adding 100 µL Streptavidin Agarose (ThermoFisher Scientific, SA10004) to each sample and incubating at 37 °C for 30 min with rotation. Beads were then washed in wash buffer five times, followed by RNA isolation. Probe sequences are shown in Table S7, Supporting Information.

### Immunofluorescence

Cells grown on coverslips were fixed with 100% ice‐cold methanol followed by incubation overnight at 4 °C with primary antibody followed by Alexa Fluor 488‐conjugated secondary antibody (Table S3, Supporting Information) in dark. Photomicrographs were collected using confocal microscopy (ZEISS LSM 900 with Airyscan 2, 63 × objective).

### Cell Viability Assay

Briefly, cells were seeded at 5 × 10^3^ per well in 96‐well plates overnight before experimental treatments. MTS solution (VisionBlue Quick Cell Viability Fluorometric Assay Kit, K303‐2500) was added according to the manufacturer's instructions and incubated at 37 °C for 1 h. The fluorescent signal was then recorded by Synergy2 multidetection microplate reader (BioTek).

### Cell Cycle Analysis

Cells were fixed by 70% ethanol at −20 °C overnight and spun down at 2500 × *g* for 5 min. Cell pellets were re‐suspended in PBS containing 0.25% Triton X‐100 and incubate on ice for 15 min. After discarding the supernatant, cell pellet was resuspended in 0.5 mL PBS containing 10 µg mL^−1^ RNase A and 20 µg mL^−1^ PI stock solution and incubated at RT in the dark for 30 min. Cells were then analyzed using a flow cytometer (FACSCanto, BD Biosciences).

### UV Irradiation

Cells were irradiated with a UV‐C dose of 10 J m^−2^ using the UVILink CL‐508 crosslinker (Cambridge, UK).

### Cell Death

The dead cells were quantitated using the FITC Annexin V Apoptosis Detection Kit I (BD Biosciences, 556 547) according to the manufacturer's instructions as described previously.^[^ ^51^ ^]^ In brief, cells in the binding buffer were incubated with Annexin V/propidium iodide (PI) for 15 min before analysis using a flow cytometer (FACSCanto II; BD Biosciences).

### BrdU Incorporation Assay

BrdU cell proliferation assays were carried out using the BrdU Cell Proliferation Assay kit (Cell Signaling). Briefly, cells were seeded at 5 × 10^3^ cells per well in 96‐well plates 24 h before treatment. Cells were then incubated with BrdU (10 mm) for 4 h and absorbance was read at 450 nm using a Synergy 2 multidetection microplate reader (BioTek, VT).

### Colony Formation

Totally, 1 × 10^3^ cells were seeded and incubated in a 6‐well plate. After 2 weeks of culture, the cells were fixed with ice‐cold methanol, stained with crystal violet, imaged and quantified using the ImageJ‐plugin “ColonyArea.”^[^ ^52^ ^]^

### Xenograft Mouse Model

Cells expressing inducible LIMp27 shRNAs were subcutaneously injected into the dorsal flanks of 4 week‐old female nude mice (6 mice per group, Changzhou Cavens Laboratory Animal Co. Ltd.). Tumor growth was measured every 3 days using a calliper. Mice were sacrificed, and tumors were excised and measured at the endpoint. Studies on animals were conducted in accordance with relevant guidelines and regulations and were approved by the Animal Research Ethics Committee of Huai'an No.1 People's Hospital (DW‐P‐2021‐005‐001). All mice were housed in a temperature‐controlled room (21—23 °C) with 40–60% humidity and a light/dark cycle of 12 h/12 h.

### Statistical Analysis

Data were normalized and presented as mean ± s.d. Sample sizes (*n*) were indicated for each statistical analysis. Statistical differences were analyzed by two‐tailed Student's *t*‐test or one‐way ANOVA test followed by Tukey's multiple comparisons. A *p*‐value less than 0.05 is statistically significant. Statistical analysis was carried out using GraphPad Prism to assess differences between experimental groups.

## Conflict of Interest

The authors declare no conflict of interest.

## Author contributions

T.L. and S.C. contributed equally to this work. T.L., S.C., X.D.Z., and L.J. designed the research; X.D.Z., L.J., T.L., F‐M.S., and R.F.T. supervised the study; T.L., S.C., X.H.Z., L.T., R.X., L.X., K.Y., S.Z., T.G., M.F.J., Y.Y.Z., Y.C.F., and H.J.T. carried out experiments; Y.W., Q.X., Y.G., and H.C. provided extra technical assistance; X.D.Z., T.L., L.J., F‐M.S., and R.F.T. wrote the manuscript. All authors commented on the manuscript.

## Supporting information

Supporting information

## Data Availability

The data that support the findings of this study are available from the corresponding author upon reasonable request.
