# Correction to “LncRNA LIMp27 Regulates the DNA Damage Response Through p27 in p53‐Defective Cancer Cells”

**DOI:** 10.1002/advs.202500857

**Published:** 2025-01-30

La T, Chen S, Zhao XH, Zhou S, Xu R, Teng L, Zhang YY, Ye K, Xu L, Guo T, Jamaluddin MF, Feng YC, Tang HJ, Wang Y, Xu Q, Gu Y, Cao H, Liu T, Thorne RF, Shao FM, Zhang XD, Jin L. LncRNA LIMp27 Regulates the DNA Damage Response through p27 in p53‐Defective Cancer Cells. *Adv Sci*, 2023 Jan 13; e2204599.

**Figure 2i**. The images of HT‐29.shCtrl untreated (‐Dox), treated with Dox (+Dox), Dox withdrawal, HT‐29.shLIMp27‐1 untreated (‐Dox), and HT‐29.shLIMp27‐2 untreated (‐Dox) were incorrect and replaced. The corrected data still demonstrate that Dox treatment inhibits cell clonogenicity associated with reduced LIMp27 expression. Moreover, the recovery of LIMp27 expression after Dox withdrawal partially restores cell clonogenicity, consistent with the conclusion.


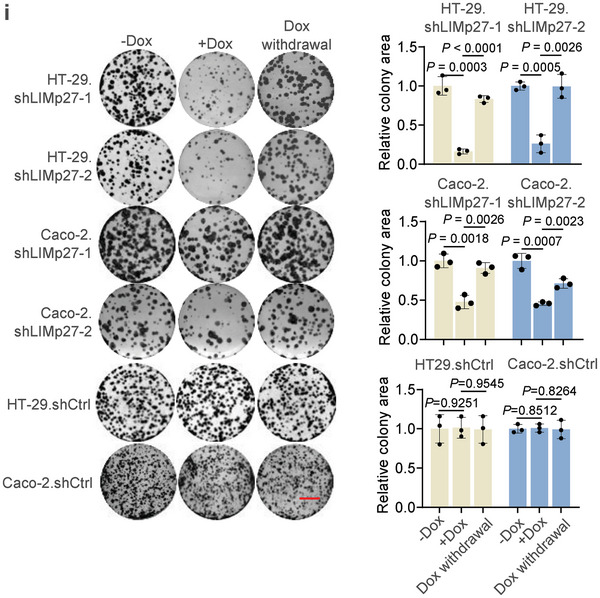


**Figure 6c**. The images of HT‐29 control siRNA, HT‐29 control siRNA plus Oxaliplatin, HT‐29 LIMp27 siRNA‐1, WiDr control siRNA, WiDr control siRNA plus Oxaliplatin, WiDr control siRNA plus IR, and WiDr LIMp27 siRNA‐1 were incorrect and replaced. The corrected figure does not alter the previous conclusion, namely that LIMp27 knockdown, in combination with oxaliplatin or IR treatment, significantly further reduces cell clonogenicity.


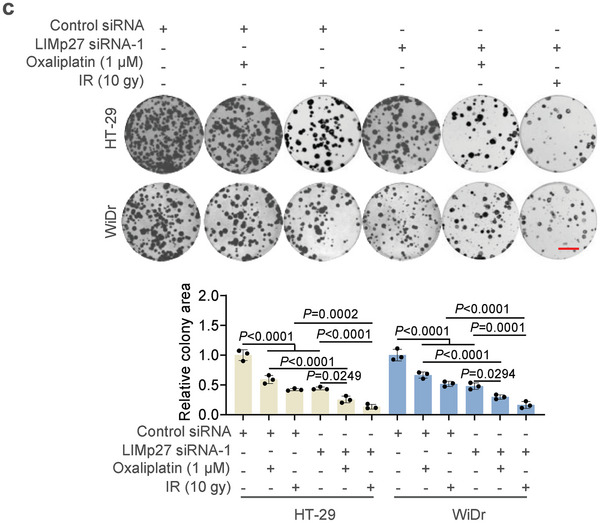


**Figure 6g**. The images of HT‐29 LIMp27 siRNA‐1 plus IR, HT‐29 LIMp27 siRNA‐1 plus p27 siRNA‐1 plus Oxaliplatin, and WiDr LIMp27 siRNA‐1 plus IR were incorrect and replaced. The corrected figure still demonstrates that the knockdown of p27 diminishes the cooperative effect of LIMp27 knockdown and oxaliplatin or IR treatment in inhibiting the viability of HT‐29 and WiDr cells. The conclusion remains unaffected.


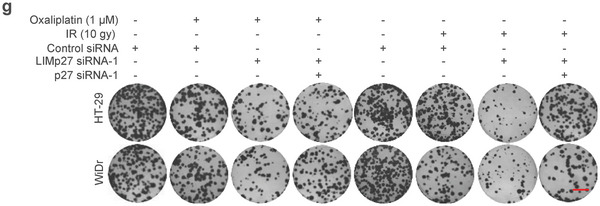


**Figure s2f**. The images of MDA‐MB‐231 LIMp27 siRNA‐1, MCF‐7 Control siRNA, MCF‐7 LIMp27 siRNA‐1, and MCF‐7 LIMp27 siRNA‐2 were incorrect and replaced. The corrected data still support the original finding that siRNA‐mediated LIMp27 knockdown markedly reduces the viability and clonogenicity in mutant p53‐expressing cells (MDA‐MB‐231 and H226), but not in wild‐type p53‐expressing cells (MCF‐7 and A549). The conclusion resmains unaffected.


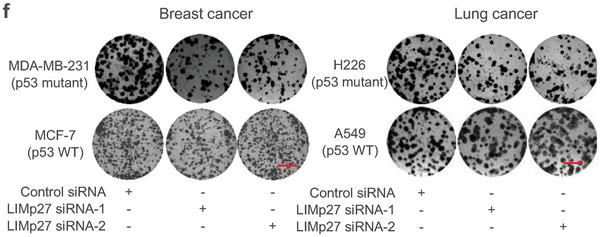


**Figure s4g**. The images of HT29 control siRNA, HT29 LIMp27 siRNA‐1, HT29 LIMp27 siRNA‐1 plus p21 siRNA‐1, WiDr control siRNA, WiDr LIMp27 siRNA‐1, and WiDr LIMp27 siRNA‐1 plus p21 siRNA‐1 were incorrect and replaced. The corrected figure corroborates the conclusion that p21 does not mitigate the reductions in clonogenicity.


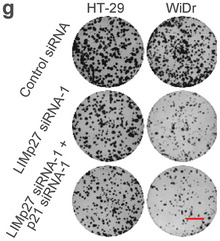


**Figure s8k**. The images of HT29 shCtrl treated with Saline only (‐Dox) and HT29 shCtrl treated with Saline plus Dox (+Dox) were incorrect and replaced. As the negative control, the corrected images confirm that Dox‐induced control shRNA, in combination with saline (vehicle control), does not affect the Ki67‐positive cell proportions. Nonetheless, the corrected whole figure upholds the original conclusion that the Dox‐induced shRNA knockdown of LIMp27, either alone or in combination with oxaliplatin, leads to a decrease in Ki67‐positive cell proportions.


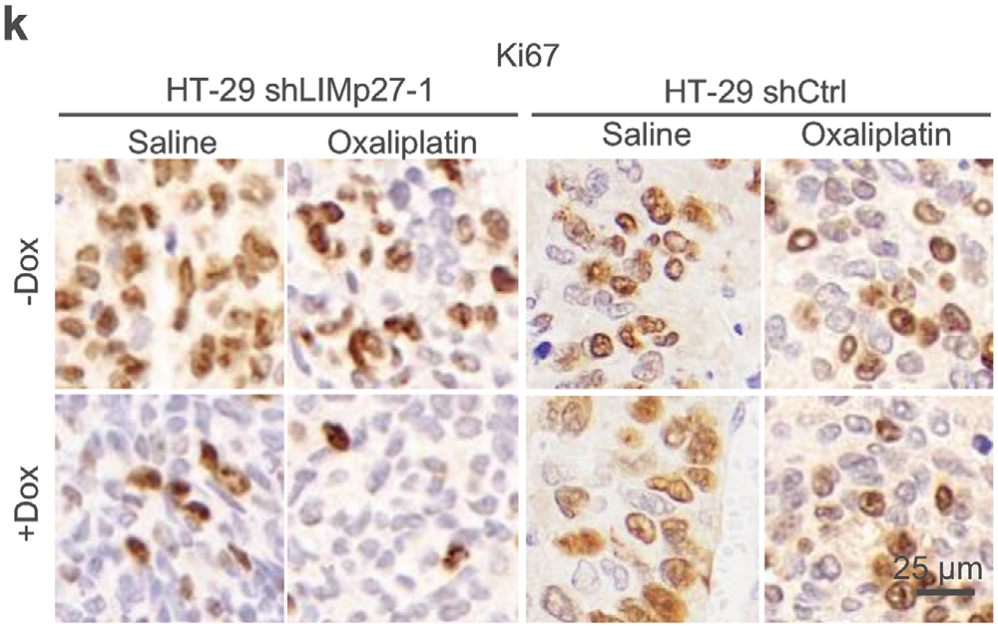


This correction does not affect the overall findings and conclusions of this paper. We apologize for these errors.

