# Supplementary material for: LncRNA LIMp27 Regulates the DNA Damage Response through p27 in p53‐Defective Cancer Cells
Source: Adv Sci (Weinh). 2023 Jan 13;10(7):2204599. doi: 10.1002/advs.202204599 (PMC9982580; doi:10.1002/advs.202204599)
Supplement: Supplementary file 1 — Supporting information [file ADVS-10-2204599-s001.pdf]

Supplementary Materials for

LncRNA LIMp27 regulates the DNA damage response through p27 in p53-defective cancer cells

Ting La,[#] Song Chen,[#] Xiao Hong Zhao, Shuai Zhou, Ran Xu, Liu Teng, Yuan Yuan Zhang, Kaihong Ye, Liang Xu, Tao Guo, Muhammad Fairuz Jamaluddin, Yu Chen Feng, Hai Jie Tang, Yanliang Wang, Qin Xu, Yue Gu, Huixia Cao, Tao Liu, Rick F. Thorne, Feng-Min Shao,* Xu Dong Zhang,* Lei Jin*

*Corresponding author. Email: Lei.Jin@newcastle.edu.au, Xu.Zhang@newcastle.edu.au or fengminshao@126.com

This PDF file includes:

Figs. S1 to S8
Tables S1 to S7

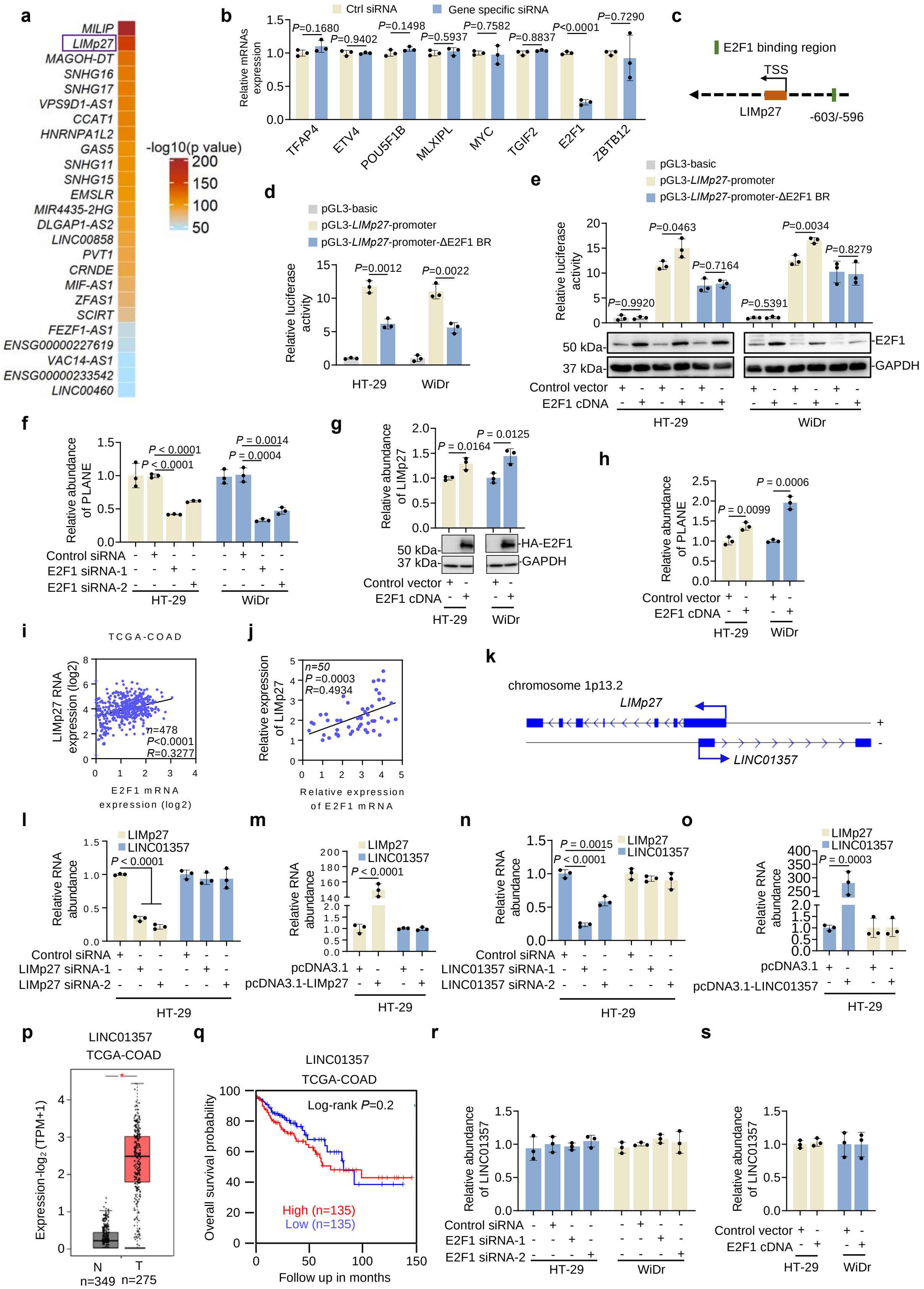
a
MILIP
LIMp27
MAGOH-DT
SNHG16
SNHG17
VPS9D1-AS1
CCAT1
HNRNPA1L2
GAS5
SNHG11
SNHG15
EMSLR
MIR4435-2HG
DLGAP1-AS2
LINC00858
PVT1
CRNDE
MIF-AS1
ZFAS1
SCIRT
FEZF1-AS1
ENSG00000227619
VAC14-AS1
ENSG00000233542
LINC00460
-log10(p value)
200
150
100
50
b
Ctrl siRNA
Gene specific siRNA
Relative mRNAs expression
P=0.1680
P=0.9402
P=0.1498
P=0.5937
P=0.7582
P=0.8837
P<0.0001
P=0.7290
TFAP4
ETV4
POU5F1B
MLXIPL
MYC
TGIF2
E2F1
ZBTB12
c
E2F1 binding region
TSS
LIMp27
-603/-596
d
pGL3-basic
pGL3-LIMp27-promoter
pGL3-LIMp27-promoter-ΔE2F1 BR
Relative luciferase activity
P=0.0012
P=0.0022
HT-29
WiDr
e
pGL3-basic
pGL3-LIMp27-promoter
pGL3-LIMp27-promoter-ΔE2F1 BR
Relative luciferase activity
P=0.9920
P=0.0463
P=0.7164
P=0.5391
P=0.0034
P=0.8279
50 kDa-
37 kDa-
E2F1
GAPDH
Control vector
E2F1 cDNA
HT-29
WiDr
f
Relative abundance of PLANE
P < 0.0001
P < 0.0001
P = 0.0014
P = 0.0004
Control siRNA
E2F1 siRNA-1
E2F1 siRNA-2
HT-29
WiDr
g
Relative abundance of LIMp27
P = 0.0164
P = 0.0125
50 kDa-
37 kDa-
HA-E2F1
GAPDH
Control vector
E2F1 cDNA
HT-29
WiDr
h
Relative abundance of PLANE
P = 0.0099
P = 0.0006
Control vector
E2F1 cDNA
HT-29
WiDr
i
TCGA-COAD
LIMp27 RNA expression (log2)
n=478
P<0.0001
R=0.3277
E2F1 mRNA expression (log2)
j
n=50
P =0.0003
R=0.4934
Relative expression of LIMp27
Relative expression of E2F1 mRNA
k
chromosome 1p13.2
LIMp27
LINC01357
l
LIMp27
LINC01357
Relative RNA abundance
P < 0.0001
Control siRNA
LIMp27 siRNA-1
LIMp27 siRNA-2
HT-29
m
LIMp27
LINC01357
Relative RNA abundance
P < 0.0001
pcDNA3.1
pcDNA3.1-LIMp27
HT-29
n
LIMp27
LINC01357
Relative RNA abundance
P = 0.0015
P < 0.0001
Control siRNA
LINC01357 siRNA-1
LINC01357 siRNA-2
HT-29
o
LIMp27
LINC01357
Relative RNA abundance
P = 0.0003
pcDNA3.1
pcDNA3.1-LINC01357
HT-29
p
LINC01357
TCGA-COAD
Expression-log2 (TPM+1)
N
T
n=349
n=275
q
LINC01357
TCGA-COAD
Log-rank P=0.2
Overall survival probability
High (n=135)
Low (n=135)
Follow up in months
r
Relative abundance of LINC01357
Control siRNA
E2F1 siRNA-1
E2F1 siRNA-2
HT-29
WiDr
s
Relative abundance of LINC01357
Control vector
E2F1 cDNA
HT-29
WiDr

Fig. S1

Fig. S1. Identification of LIMp27 as an E2F1-responsive lncRNA in COAD cells

a, Identification of a panel of lncRNAs that were commonly upregulated in at least 80% of COAD samples compared with normal colon tissues. Upregulation was defined where the lncRNA expression values in a COAD case versus the mean lncRNA expression value in normal tissue was greater than 2 × standard deviation. b, siRNA screening showed knockdown of E2F1 but not the other seven transcription factors reduced LIMp27 expression. Data are mean ± s.d.; n = 3 independent experiments, two-tailed Student's *t*-test. c, Schematic illustration of the consensus E2F1 binding region (E2F1-BR, green bar) located to the -603/-596 region of the proximal promoter of *LIMp27* gene. d, Deletion of the E2F1-BR reduced the transcriptional activity of a *LIMp27* promoter reporter construct (pGL3-*LIMp27*-promoter) in HT-29 and WiDr cells. Data are mean ± s.d.; n = 3 independent experiments, two-tailed Student's *t*-test. e, Overexpression of E2F1 enhanced the transcriptional activity of a *LIMp27* reporter construct with the intact E2F1 binding region (BR) (pGL3-*LIMp27*-promoter) but did not affect the activity of a construct with the E2F1-BR deleted (pGL3-*LIMp27*-promoter-ΔE2F1-BR) in HT-29 and WiDr cells. Data are mean ± s.d.; n = 3 independent experiments, two-tailed Student's *t*-test. f, E2F1 silencing downregulated PLANE expression in HT-29 and WiDr cells. Data are mean ± s.d.; n = 3 independent experiments, one-way ANOVA followed by Tukey's multiple comparisons test. g, h, Overexpression of E2F1 caused upregulation of LIMp27(g) and PLANE (h) in HT-29 and WiDr cells. Data are mean ± s.d. or representatives; n = 3 independent experiments, two-tailed Student's *t*-test. i, Linear regression analysis of the relationship between LIMp27 and E2F1 mRNA expression in the COAD dataset derived from the TCGA. Two-tailed Pearson correlation coefficient test. j, Linear regression analysis of the relationship between LIMp27 and E2F1 mRNA expression in a cohort of freshly isolated COAD samples. n = 50 biologically independent samples. Two-tailed Pearson correlation coefficient test. k, Schematic illustration of the genomic location of the *LIMp27* and *LINC01357* genes. l, m, Knockdown (l) or overexpression (m) of LIMp27 did not impinge on LINC01357 expression in HT-29 cells. Data are mean ± s.d.; n = 3 independent experiments, one-way ANOVA followed by Tukey's multiple comparisons test. n, o, Knockdown (n) or overexpression (o) of LINC01357 did not impinge on LIMp27 expression in HT-29 and WiDr cells. Data are mean ± s.d.; n = 3 independent experiments, one-way ANOVA followed by Tukey's multiple comparisons test. p, Comparison of LINC01357 expression between COAD and normal colon tissues (merge TCGA normal and GTEx data) in the COAD dataset derived from the TCGA dataset using GEPIA2 (http://gepia2.cancer-pku.cn/#index). q, Kaplan-Meier analysis of the probability of overall survival of COAD (n=270) patients derived from the TCGA dataset using the median of LINC01357 levels as the cutoff. The figure is generated using GEPIA2 (http://gepia2.cancer-pku.cn/#index). r, E2F1 silencing has no effect on LINC01357 expression in HT-29 and WiDr cells. Data are mean ± s.d.; n = 3 independent experiments, one-way ANOVA followed by Tukey's multiple comparisons test. s, Overexpression of E2F1 did not impinge on LINC01357 expression in HT-29 and WiDr cells. Data are mean ± s.d.; n = 3 independent experiments, two-tailed Student's *t*-test.

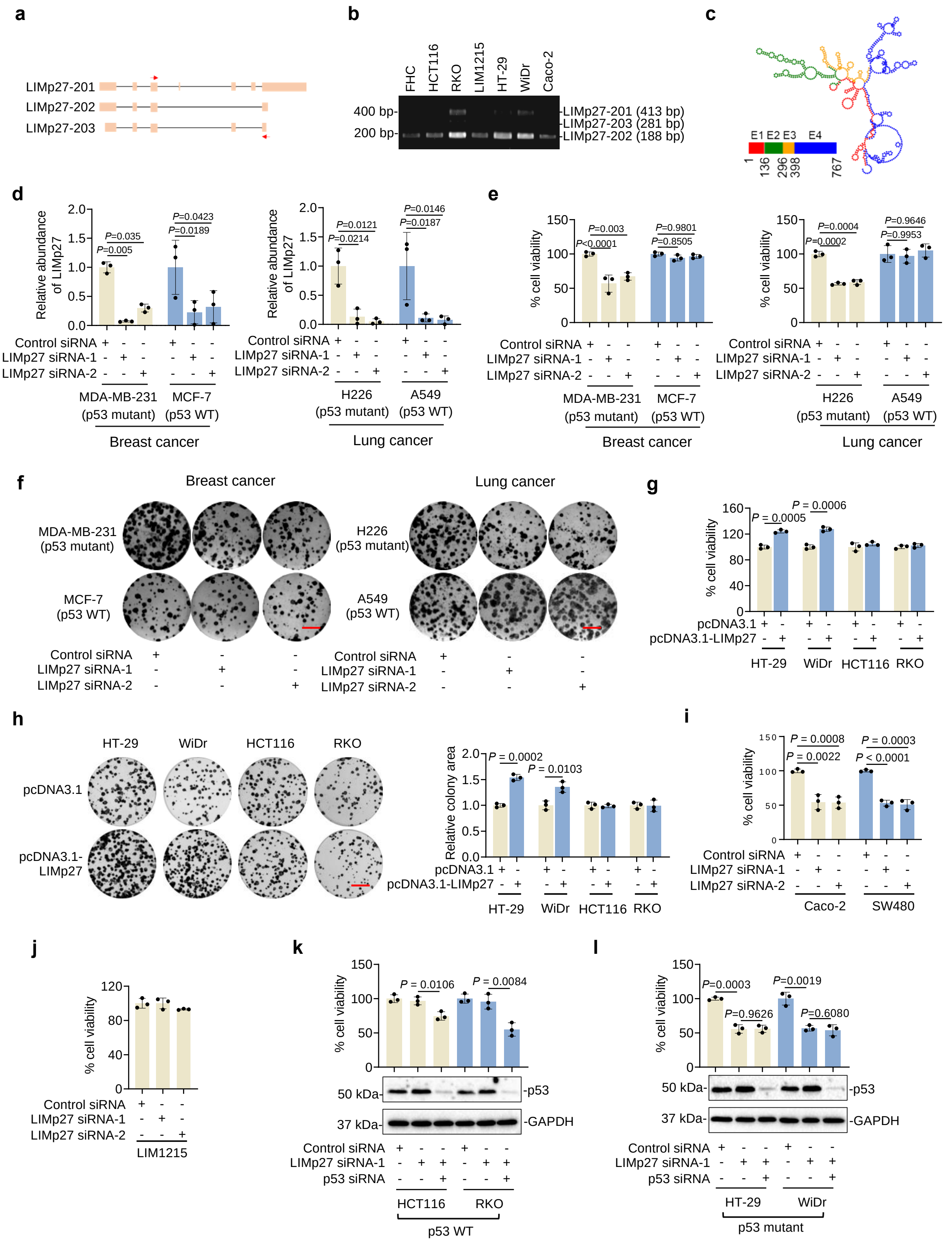
a
LIMp27-201
LIMp27-202
LIMp27-203
b
FHC
HCT116
RKO
LIM1215
HT-29
WiDr
Caco-2
400 bp-
200 bp-
-LIMp27-201 (413 bp)
-LIMp27-203 (281 bp)
-LIMp27-202 (188 bp)
c
E1 E2 E3 E4
1
136
296
398
767
d
Relative abundance of LIMp27
P=0.035
P=0.005
P=0.0423
P=0.0189
P=0.0121
P=0.0214
P=0.0146
P=0.0187
Control siRNA
LIMp27 siRNA-1
LIMp27 siRNA-2
MDA-MB-231 (p53 mutant)
MCF-7 (p53 WT)
Breast cancer
H226 (p53 mutant)
A549 (p53 WT)
Lung cancer
e
% cell viability
P=0.003
P<0.0001
P=0.9801
P=0.8505
P=0.0004
P=0.0002
P=0.9646
P=0.9953
f
Breast cancer
Lung cancer
MDA-MB-231 (p53 mutant)
MCF-7 (p53 WT)
H226 (p53 mutant)
A549 (p53 WT)
g
P = 0.0005
P = 0.0006
pcDNA3.1
pcDNA3.1-LIMp27
HT-29
WiDr
HCT116
RKO
h
pcDNA3.1-LIMp27
Relative colony area
P = 0.0002
P = 0.0103
i
P = 0.0008
P = 0.0022
P = 0.0003
P < 0.0001
Caco-2
SW480
j
LIM1215
k
P = 0.0106
P = 0.0084
50 kDa-
-p53
37 kDa-
-GAPDH
p53 siRNA
p53 WT
l
P=0.0003
P=0.9626
P=0.0019
P=0.6080
p53 mutant

Fig. S2

Fig. S2. LIMp27 selectively supports the viability of p53-defective cancer cells
a, Schematic illustration of the three annotated LIMp27 transcripts. Filled boxes represent exons. The paired primers used to distinguish each transcript were depicted in red. b, PCR analysis showing that the LIMp27-202 was markedly more abundant than other transcripts in the indicated cancer cell lines. Data are representatives of 3 independent experiments. c, The secondary structure model of LIMp27-202 was predicted based on minimum free energy algorithm. d-f, SiRNA knockdown of LIMp27 (d) inhibited cell viability (e) and clonogenicity (f) in MDA-MB-231 and H226 (p53 mutant) but not in MCF-7 and A549 (p53 WT) cell lines. Data are mean ± s.d. or representatives; n = 3 independent experiments, one-way ANOVA followed by Tukey's multiple comparison test. Scale bar, 1 cm. g, h, Overexpression of LIMp27 promoted the viability (g) and clonogenicity (h) in HT-29 and WiDr but not in HCT116 and RKO COAD cells. Data are mean ± s.d. or representatives; n = 3 independent experiments, two-tailed Student's *t*-test. Scale bar, 1 cm. i, j, SiRNA knockdown of LIMp27 inhibited cell viability of additional mutant p53 cell lines Caco-2 and SW480 (i) but not wild-type p53 cell line LIM1215 (j). Data are mean ± s.d; n = 3 independent experiments, one-way ANOVA followed by Tukey's multiple comparison test. k, Knockdown of p53 rendered HCT116 and RKO cells susceptible to LIMp27 knockdown-induced inhibition of cell viability. WT: wild-type. Data are mean ± s.d. or representatives; n = 3 independent experiments, one-way ANOVA followed by Tukey's multiple comparisons test. l, Knockdown of mutant p53 in HT-29 and WiDr cells did not impinge on the inhibition of cell viability caused by LIMp27 knockdown. Data are mean ± s.d. or representatives; n = 3 independent experiments, one-way ANOVA followed by Tukey's multiple comparisons test.

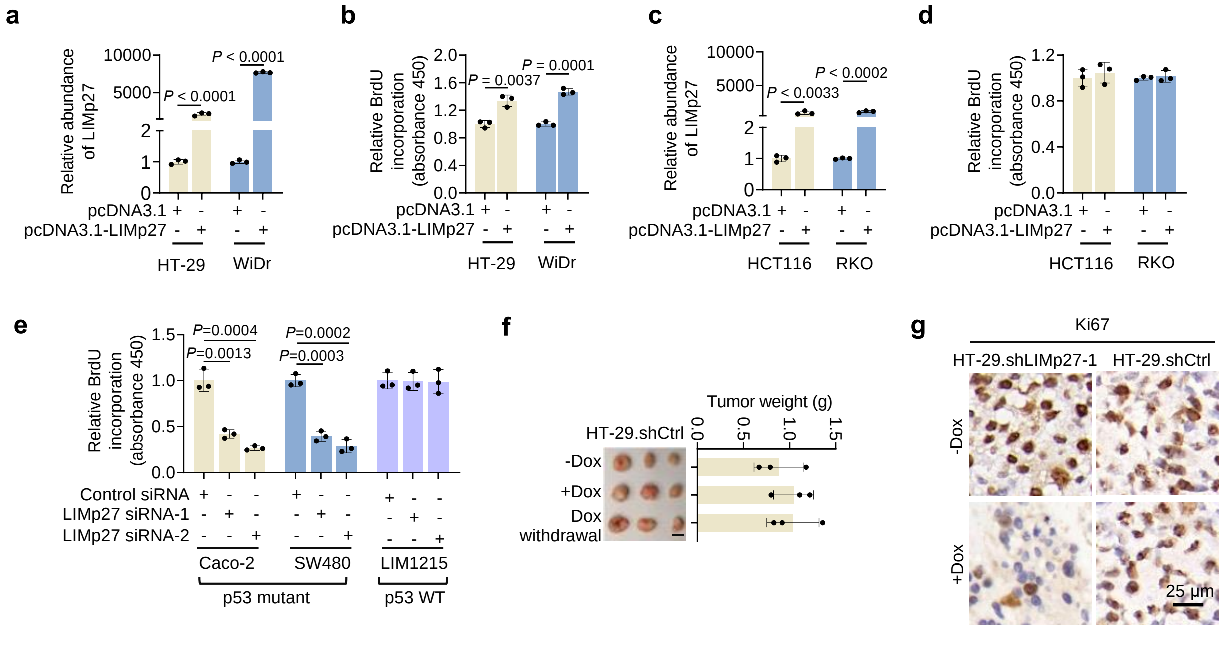

Fig. S3. LIMp27 promotes p53-mutant COAD cell proliferation and tumorigenicity.
a-d, Overexpression of LIMp27 (a, c) promoted cell proliferation as shown by increased BrdU incorporation (b, d) in HT-29 and WiDr cells carrying mutant p53 (b) but not in HCT116 and RKO cells harboring wild-type p53 (d). Data are mean ± s.d.; n = 3 independent experiments, two-tailed Student's *t*-test. e, SiRNA knockdown of LIMp27 inhibited cell proliferation as shown by decreased BrdU incorporation in Caco-2 and SW480 cells carrying mutant p53 but not in LIM1215 cells harboring wild-type p53. Data are mean ± s.d.; n = 3 independent experiments, two-tailed Student's *t*-test. f, Representative photographs & tumor weights showing Dox treatment/withdrawal did not alter HT-29.shCtrl xenograft growth in nu/nu mice. Data are mean ± s.d.; n = 3 mice per group. Dox: 2mg/mL supplemented with 10 mg/mL sucrose in drinking water. Scale bar, 1 cm. g, Representative microphotographs of immunohistochemistry staining using an antibody against Ki-67 in HT-29.shLIMp27 and HT-29.shCtrl mouse xenografts with or without treatment with Dox. n=6 mice per group. Scale bar, 25 μm.

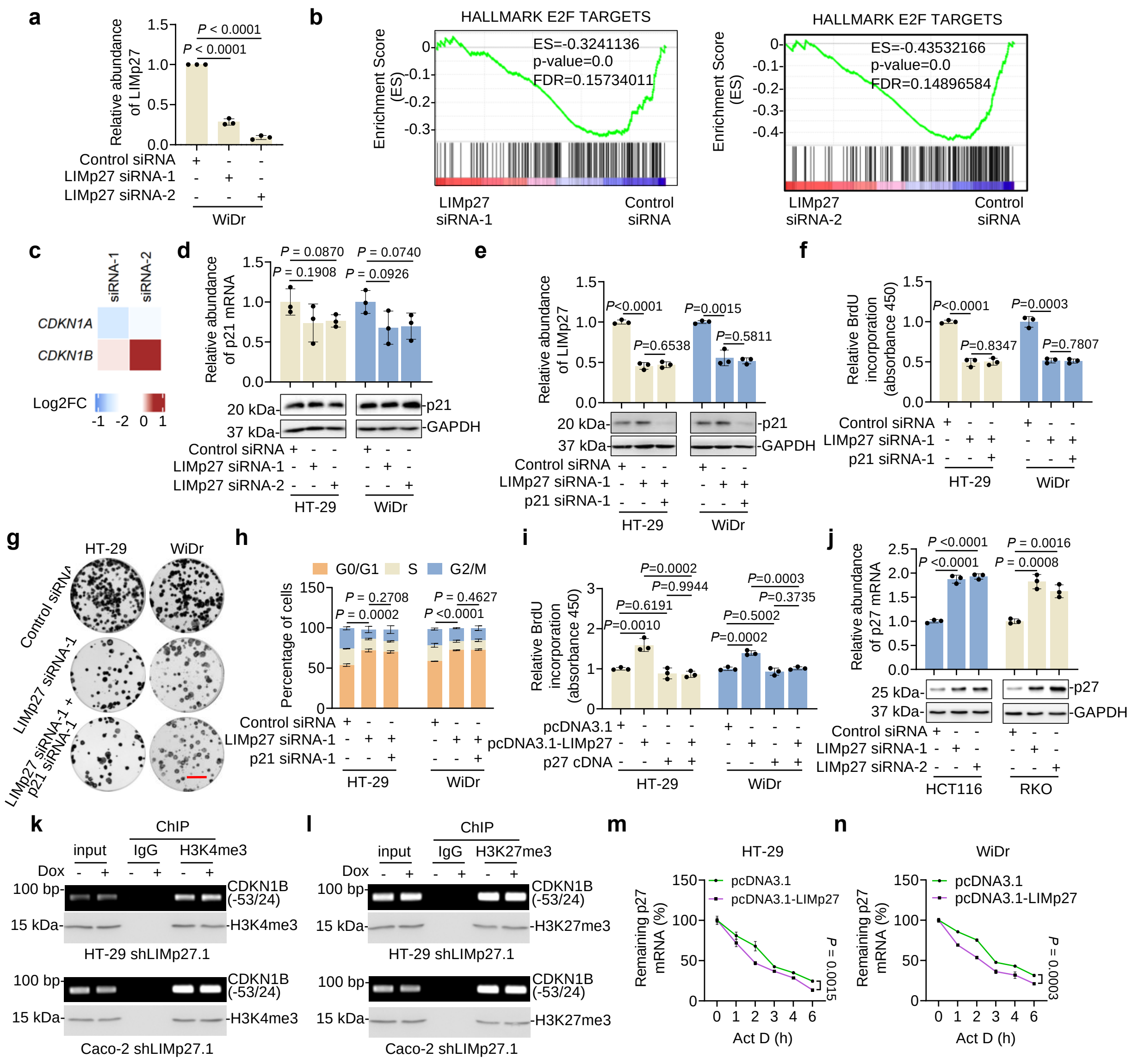

a
Relative abundance of LIMp27
P < 0.0001
Control siRNA
LIMp27 siRNA-1
LIMp27 siRNA-2
WiDr
b
HALLMARK E2F TARGETS
Enrichment Score (ES)
ES=-0.3241136
p-value=0.0
FDR=0.15734011
ES=-0.43532166
FDR=0.14896584
LIMp27 siRNA-1
LIMp27 siRNA-2
Control siRNA
c
siRNA-1
siRNA-2
CDKN1A
CDKN1B
Log2FC
d
Relative abundance of p21 mRNA
P = 0.0870
P = 0.1908
P = 0.0740
P = 0.0926
p21
GAPDH
HT-29
WiDr
e
P<0.0001
P=0.6538
P=0.0015
P=0.5811
p21 siRNA-1
f
Relative BrdU incorporation (absorbance 450)
P=0.8347
P=0.0003
P=0.7807
g
Control siRNA
LIMp27 siRNA-1
LIMp27 siRNA-1 + p21 siRNA-1
h
G0/G1
S
G2/M
Percentage of cells
P = 0.2708
P = 0.0002
P = 0.4627
P <0.0001
i
P=0.0002
P=0.9944
P=0.6191
P=0.0010
P=0.0003
P=0.3735
P=0.5002
pcDNA3.1
pcDNA3.1-LIMp27
p27 cDNA
j
Relative abundance of p27 mRNA
P = 0.0016
P = 0.0008
p27
HCT116
RKO
k
ChIP
input
IgG
H3K4me3
Dox
CDKN1B (-53/24)
HT-29 shLIMp27.1
Caco-2 shLIMp27.1
l
H3K27me3
m
Remaining p27 mRNA (%)
Act D (h)
P = 0.0015
n
P = 0.0003

Fig. S4

Fig. S4. LIMp27 represses p27 expression through destabilizing its mRNA

a, LIMp27 knockdown efficiency in WiDr cells was confirmed using qPCR and subjected to RNA-seq. Data are mean ± s.d.; n = 3 independent experiments, one-way ANOVA followed by Tukey's multiple comparisons test. b, Gene Set Enrichment Analysis (GSEA) of RNA-seq data from WiDr cells with or without LIMp27 knockdown. n=3 experimental repeats. ES, enrichment score; FDR, false-discovery rate. c, Heatmap showing fold changes of CDKN1A and CDKN1B mRNA expression derived from RNA-seq of WiDr cells with or without LIMp27 knockdown. n = 3 biological repeats. d, LIMp27 knockdown did not upregulate the expression of p21. Data are mean ± s.d. or representatives; n = 3 independent experiments, one-way ANOVA followed by Tukey's multiple comparison test. e-h, LIMp27 knockdown-induced upregulation of p27 expression (e), inhibition of BrdU incorporation (f) and clonogenicity (g), and G0/G1 phase cell cycle arrest (h) were not rescued by p21 co-knockdown in HT-29 and WiDr cells. Data are representatives or mean ± s.d.; n = 3 independent experiments, One-way ANOVA followed by Tukey's multiple comparisons test. Scale bar, 1 cm. i, The upregulation of BrdU incorporation caused by LIMp27 overexpression were diminished by p27 overexpression in HT-29 and WiDr cells. Data are mean ± s.d.; n = 3 independent experiments, One-way ANOVA followed by Tukey's multiple comparisons test. j, LIMp27 knockdown upregulated p27 expression at both mRNA and protein levels in HCT116 and RKO cells. Data are mean ± s.d. or representatives; n = 3 independent experiments, one-way ANOVA followed by Tukey's multiple comparison test. k, l, Induced knockdown of LIMp27 did not affect the enrichment of the transcriptional activation marker H3K4me3 (k) and the transcriptional repression marker H3K27me3 (l) to the *CDKN1B* promoter in HT-29 and Caco-2 cells as shown using chromatin immunoprecipitation (ChIP) assays. Data are representatives; n = 3 independent experiments. m, n, Total RNA from HT-29 (m) and WiDr (n) cells transfected with indicated plasmids and treated with Actinomycin D (Act D, 1 μg/mL) for indicated periods were subjected to qPCR. Data are mean ± s.d.; n = 3 independent experiments, two-tailed Student's *t*-test.

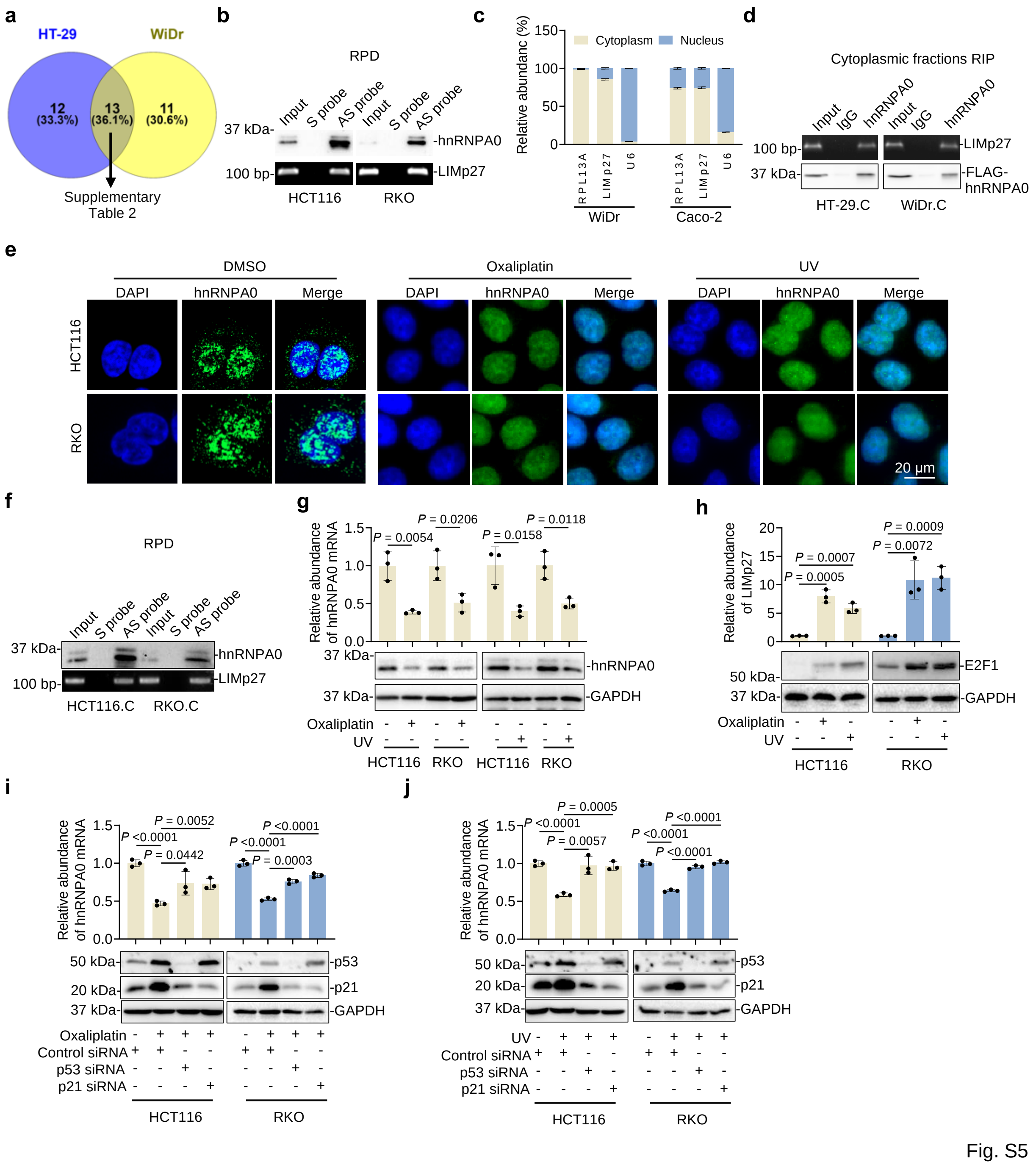
a
HT-29
WiDr
12 (33.3%)
13 (36.1%)
11 (30.6%)
Supplementary Table 2
b
RPD
Input S probe AS probe Input S probe AS probe
37 kDa-
-hnRNPA0
100 bp-
-LIMp27
HCT116
RKO
c
Relative abundanc (%)
Cytoplasm
Nucleus
RPL13A LIMp27 U6
WiDr
Caco-2
d
Cytoplasmic fractions RIP
Input IgG hnRNPA0
100 bp-
-LIMp27
37 kDa-
-FLAG-hnRNPA0
HT-29.C
WiDr.C
e
DMSO
Oxaliplatin
UV
DAPI
hnRNPA0
Merge
HCT116
RKO
20 μm
f
RPD
HCT116.C
RKO.C
g
Relative abundance of hnRNPA0 mRNA
P = 0.0054
P = 0.0206
P = 0.0158
P = 0.0118
-GAPDH
Oxaliplatin
UV
h
Relative abundance of LIMp27
P = 0.0007
P = 0.0005
P = 0.0009
P = 0.0072
-E2F1
i
P = 0.0052
P <0.0001
P = 0.0442
P <0.0001
P = 0.0003
50 kDa-
-p53
20 kDa-
-p21
Control siRNA
p53 siRNA
p21 siRNA
j
P = 0.0005
P = 0.0057
P <0.0001
Fig. S5

Fig. S5. LIMp27 interacts with cytoplasmic hnRNPA0

a, 13 common proteins interacting with LIMp27 in both HT-29 and WiDr cells were identified by mass spectrometry. The detailed list was shown in Supplementary Table 2. b, hnRNPA0 was co-pulled down with LIMp27 in HCT116 and RKO cells as shown in RNA pulldown (RPD) assays. S, sense; AS, antisense. Data are representatives of three independent experiments. c, qPCR analysis showed that LIMp27 is mainly located in cytoplasmic rather than nuclear fractions in HT-29 and Caco-2 cells. U6 and RPL13A were included as controls for nuclear and cytoplasmic fractions, respectively. Data are mean ± s.d.; n = 3 independent experiments. d, LIMp27 was co-precipitated with hnRNPA0 in the cytoplasmic fraction of HT-29 and WiDr cells as shown in RNA immunoprecipitation (RIP) assays. C: cytoplasm. Data are representatives of three independent experiments. e, Immunofluorescence staining of hnRNPA0 in HCT116 and RKO cell lines treated with or without oxaliplatin (1 μM) or UV irradiation (10 J/m2). Scale bar, 20 μm. f, hnRNPA0 was co-pulled down with LIMp27 in the cytoplasmic fraction of HCT116 and RKO cells as shown in RNA pulldown assays. S, sense; AS, antisense; C: cytoplasm. Data are representatives of three independent experiments. g, p53-WT HCT116 and RKO were treated with or without 1 μM oxaliplatin treatment or UV irradiation (10 J/m^2). hnRNPA0 protein expression was measured by western blotting. hnRNPA0 mRNA expression was measured by qPCR. Data are mean ± s.d. or representatives; n = 3 independent experiments, two-tailed Student's *t*-test. h, HCT116 and RKO cells treated with or without oxaliplatin (1 μM) or UV irradiation (10 J/m2) were subjected to Western blotting and qPCR. Data are mean ± s.d. or representatives; n = 3 independent experiments, two-tailed Student's *t*-test. i, j, HT-29 and WiDr cells transfected with indicated siRNAs and treated with or without oxaliplatin (1 μM) for 24 hours (i) or UV irradiation (10 J/m^2, j) were subjected to qPCR. Data are representatives or mean ± s.d.; n = 3 independent experiments, One-way ANOVA followed by Tukey's multiple comparisons test.

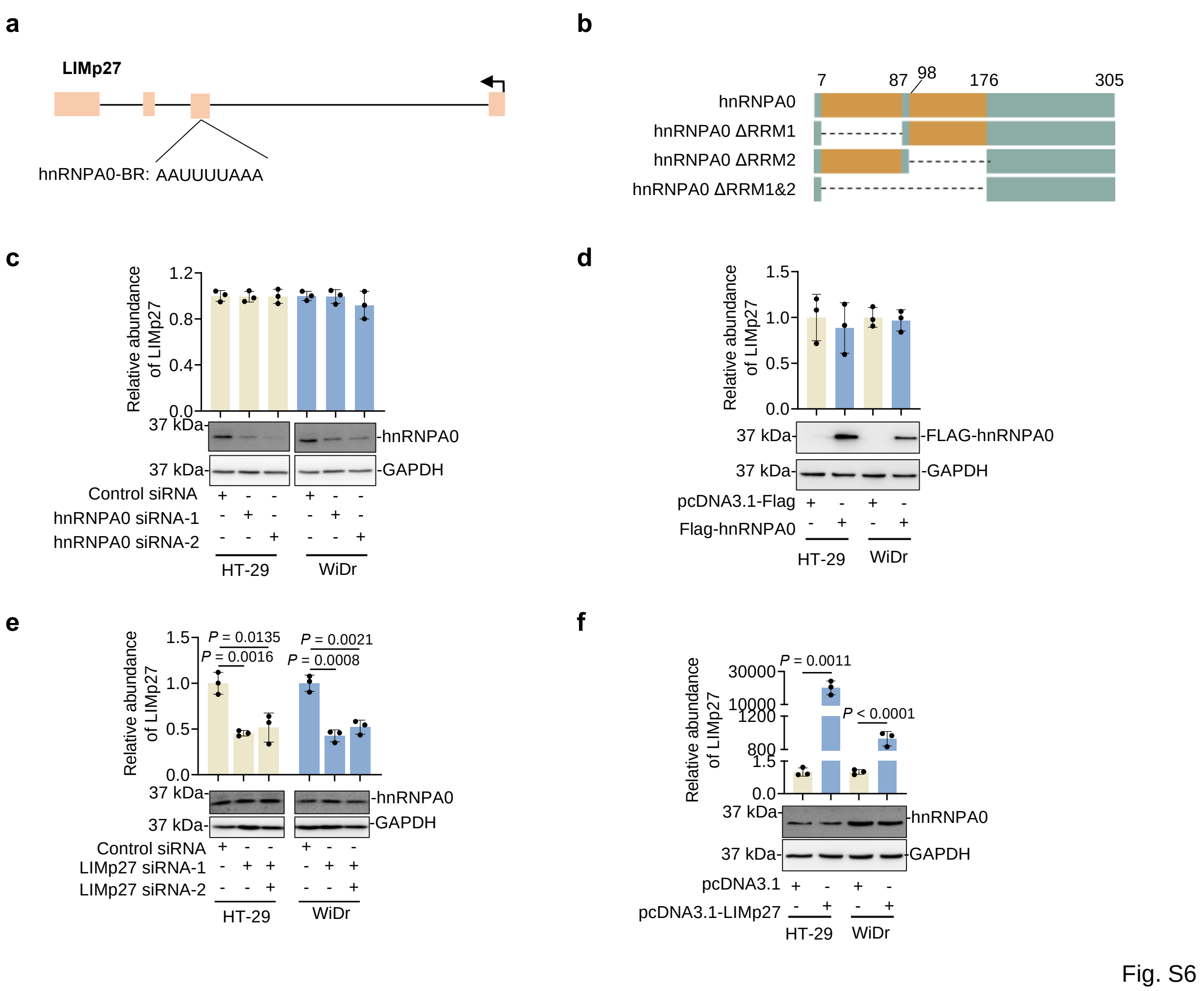

Fig. S6. The interaction between LIMp27 and hnRNPA0 does not alter their expression levels

a, Schematic illustration of LIMp27 and its putative AU-rich hnRNPA0 binding region (AAUUUUAAA). b, Schematic illustration of full-length hnRNPA0 and the hnRNPA0 mutants with individual or both RNA recognition motifs (RRM1and RRM2) deleted. c, d, hnRNPA0 silencing (c) and overexpression (d) did not alter the expression of LIMp27 in HT-29 and WiDr cells. Data are mean ± s.d. or representatives; n = 3 independent experiments, two-tailed Student's *t*-test. e, f, LIMp27 silencing (e) and overexpression (f) did not alter the expression of hnRNPA0 in HT-29 and WiDr cells. Data are mean ± s.d. or representatives; n = 3 independent experiments, two-tailed Student's *t*-test.

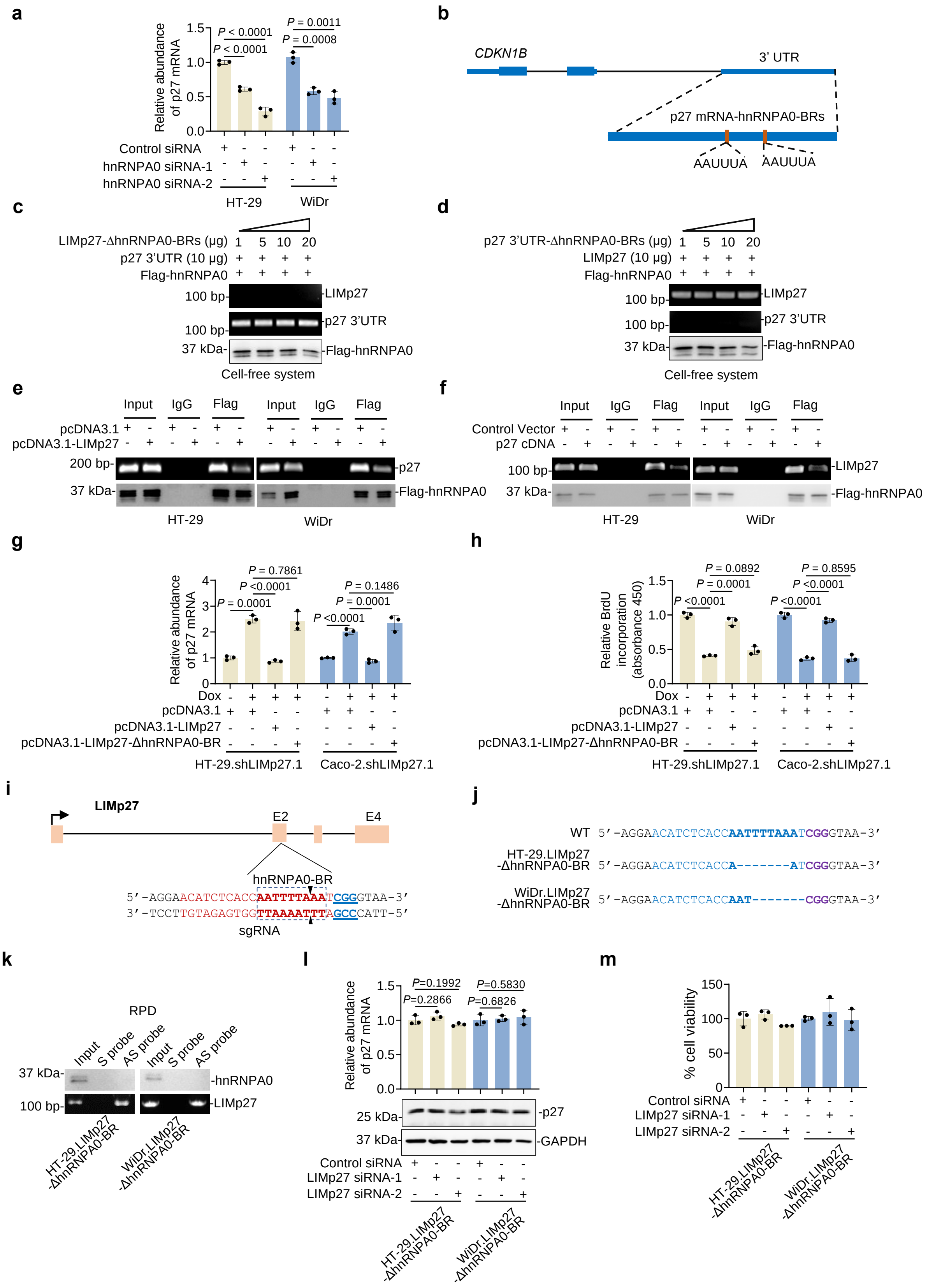
a
Relative abundance of p27 mRNA
P < 0.0001
P < 0.0001
P = 0.0011
P = 0.0008
Control siRNA
hnRNPA0 siRNA-1
hnRNPA0 siRNA-2
HT-29
WiDr
b
CDKN1B
3' UTR
p27 mRNA-hnRNPA0-BRs
AAUUUA
AAUUUA
c
LIMp27-ΔhnRNPA0-BRs (μg) 1 5 10 20
p27 3'UTR (10 μg)
Flag-hnRNPA0
100 bp-
-LIMp27
-p27 3'UTR
37 kDa-
-Flag-hnRNPA0
Cell-free system
d
p27 3'UTR-ΔhnRNPA0-BRs (μg) 1 5 10 20
LIMp27 (10 μg)
Flag-hnRNPA0
-LIMp27
-p27 3'UTR
-Flag-hnRNPA0
Cell-free system
e
Input IgG Flag Input IgG Flag
pcDNA3.1
pcDNA3.1-LIMp27
200 bp-
-p27
37 kDa-
-Flag-hnRNPA0
HT-29
WiDr
f
Control Vector
p27 cDNA
-LIMp27
-Flag-hnRNPA0
g
Relative abundance of p27 mRNA
P = 0.7861
P <0.0001
P = 0.0001
P = 0.1486
P = 0.0001
P <0.0001
Dox
pcDNA3.1
pcDNA3.1-LIMp27
pcDNA3.1-LIMp27-ΔhnRNPA0-BR
HT-29.shLIMp27.1
Caco-2.shLIMp27.1
h
Relative BrdU incorporation (absorbance 450)
P = 0.0892
P = 0.0001
P <0.0001
P = 0.8595
P <0.0001
P <0.0001
i
LIMp27
E2
E4
hnRNPA0-BR
5'-AGGAACATCTCACCAATTTTAAATCGGGTAA-3'
3'-TCCTTGTAGAGTGGTTAAAATTTAGCCCATT-5'
sgRNA
j
WT 5'-AGGAACATCTCACCAATTTTAAATCGGGTAA-3'
HT-29.LIMp27 -ΔhnRNPA0-BR 5'-AGGAACATCTCACCA-------ATCGGGTAA-3'
WiDr.LIMp27 -ΔhnRNPA0-BR 5'-AGGAACATCTCACCAAT-------CGGGTAA-3'
k
RPD
Input S probe AS probe Input S probe AS probe
37 kDa-
-hnRNPA0
100 bp-
-LIMp27
HT-29.LIMp27 -ΔhnRNPA0-BR
WiDr.LIMp27 -ΔhnRNPA0-BR
l
Relative abundance of p27 mRNA
P=0.1992
P=0.2866
P=0.5830
P=0.6826
25 kDa-
-p27
37 kDa-
-GAPDH
Control siRNA
LIMp27 siRNA-1
LIMp27 siRNA-2
HT-29.LIMp27 -ΔhnRNPA0-BR
WiDr.LIMp27 -ΔhnRNPA0-BR
m
% cell viability
Control siRNA
LIMp27 siRNA-1
LIMp27 siRNA-2
HT-29.LIMp27 -ΔhnRNPA0-BR
WiDr.LIMp27 -ΔhnRNPA0-BR

Fig. S7

Fig. S7. LIMp27 promotes p27 mRNA degradation through competitively binding to cytoplasmic hnRNPA0

a, hnRNPA0 knockdown downregulated p27 mRNA expression levels. Data are mean ± s.d; n = 3 independent experiments, one-way ANOVA followed by Tukey's multiple comparison test. b, Schematic illustration of p27 mRNA and the two AU-rich hnRNPA0 binding regions (hnRNPA0-BRs, AAUUUA) located at its 3'UTR. c, Increasing amount of *in vitro*-synthesized LIMp27 with hnRNPA0-BR (1-20 μg) deleted were incubated with certain amounts of the 3'UTR of the p27 mRNA (10 μg) and recombinant hnRNPA0, and then were subjected to RIP assay. Data are representatives of three independent experiments. d, Increasing amount of *in vitro*-synthesized p27 3'UTR with hnRNPA0-BRs (1-20 μg) deleted were incubated with certain amounts of the LIMp27 (10 μg) and recombinant hnRNPA0, and then were subjected to RIP assay. Data are representatives of three independent experiments. e, HT-29 and WiDr cells with or without LIMp27 overexpression were subjected to RIP assay. Data are representatives of three independent experiments. f, HT-29 and WiDr cells with or without p27 overexpression were subjected to RIP assay. Data are representatives of three independent experiments. g, h, HT-29.shLIMp27 and Caco-2.shLIMp27 cells transfected with shRNA-resistant LIMp27 but not shRNA-resistant LIMp27 with hnRNPA0-BR deleted diminished the upregulation of p27 expression (g) and inhibition of cell proliferation (h) caused by inducible knockdown of endogenous LIMp27 by Dox. Data are mean ± s.d.; n = 3 independent experiments, one-way ANOVA followed by Tukey's multiple comparison test. i, Schematic illustration of sgRNA targeting *LIM27*-hnRNPA0-BR locus. The Protospacer Adjacent Motif (PAM) sequence was highlighted in blue; The sgRNA targeting sequence was highlighted in red; The predicted cutting site for sgRNA was indicated by black arrows. j, hnRNPA0-BR wild-type (WT) and deleted mutant (ΔhnRNPA0-BR) alleles in LIMp27 exon2 (E2) region generated by CRISPR/Cas9 were examined by Sanger sequencing. k, hnRNPA0 was not co-pulled down with endogenous LIMp27 lacking hnRNPA0-BR. S, sense; AS, antisense. Data are representatives of three independent experiments. l, m, LIMp27 siRNA knockdown did not alter p27 expression (l) and cell viability (m) of HT-29.LIMp27-ΔhnRNPA0-BR and WiDr.LIMp27-ΔhnRNPA0-BR. Data are mean ± s.d.; n = 3 independent experiments, one-way ANOVA followed by Tukey's multiple comparison test.

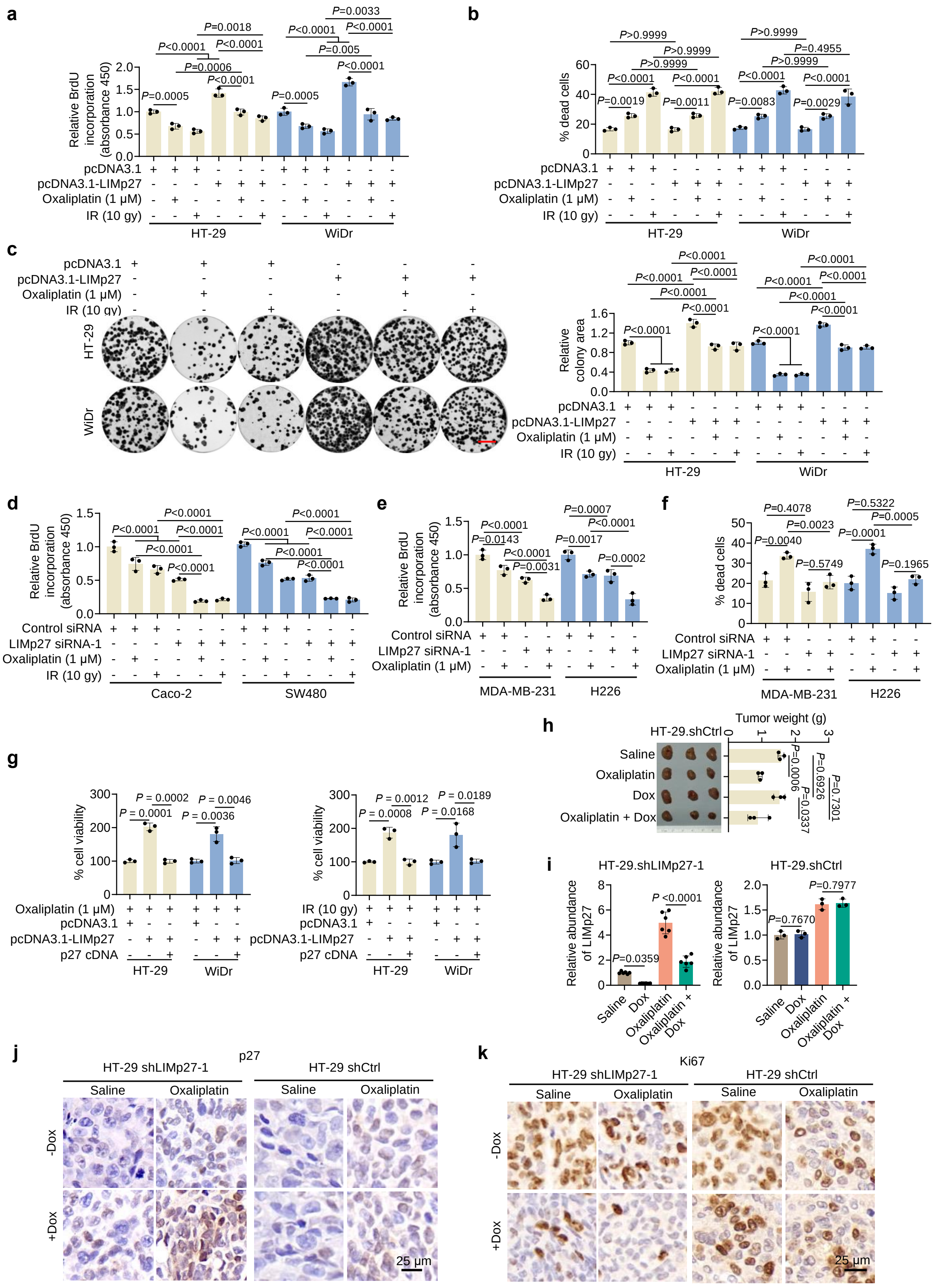

a
b
c
d
e
f
g
h
i
j
k
pcDNA3.1
pcDNA3.1-LIMp27
Oxaliplatin (1 μM)
IR (10 gy)
HT-29
WiDr
Relative BrdU incorporation (absorbance 450)
% dead cells
Relative colony area
Control siRNA
LIMp27 siRNA-1
Caco-2
SW480
MDA-MB-231
H226
% cell viability
p27 cDNA
Tumor weight (g)
HT-29.shCtrl
Saline
Oxaliplatin
Dox
Oxaliplatin + Dox
HT-29.shLIMp27-1
Relative abundance of LIMp27
p27
HT-29 shLIMp27-1
HT-29 shCtrl
-Dox
+Dox
25 μm
Ki67

Fig. S8

Fig. S8. LIMp27 regulates mutant p53 COAD cell responses to DNA-damaging therapeutics

a-c, HT-29 and WiDr cells with or without LIMp27 overexpression were treated with or without oxaliplatin (1 μM) for 24 hours or IR (10 gy), and then were subjected to BrdU incorporation (a), cell death assay (b) and clonogenicity assay (c). IR: ionizing radiation. Data are mean ± s.d. or representatives; n = 3 independent experiments, One-way ANOVA followed by Tukey's multiple comparisons test. d, Caco-2 and SW480 cells transfected with LIMp27 siRNA were treated with or without oxaliplatin (1 μM) for 24 hours or IR (10 gy), and then were subjected to BrdU incorporation. IR: ionizing radiation. Data are mean ± s.d.; n = 3 independent experiments, One-way ANOVA followed by Tukey's multiple comparisons test. e, f, MDA-MB-231 and H226 cells transfected with LIMp27 siRNA were treated with or without oxaliplatin (1 μM) for 24 hours, and then were subjected to BrdU incorporation (e) and cell death assay (f). Data are mean ± s.d.; n = 3 independent experiments, One-way ANOVA followed by Tukey's multiple comparisons test. g, LIMp27 overexpression promoted cell viability, which was reversed by overexpression of p27 in HT-29 and WiDr cells exposed to oxaliplatin (1 μM, left) and IR (10 gy, right). Data are mean ± s.d.; n = 3 independent experiments, One-way ANOVA followed by Tukey's multiple comparisons test. h, Representative photographs & tumor weights showing Dox did not alter HT-29.shCtrl xenograft growth with and without treatment with oxaliplatin (5 mg/kg by intraperitoneal injection, twice a week). Data are mean ± s.d.; n = 3 mice per group, one-way ANOVA followed by Tukey's multiple comparison test. DOX: 2mg/mL supplemented with 10 mg/mL sucrose in drinking water. i-k, The expression levels of LIMp27 (i), p27 (j), and Ki67 (k) in HT-29.shLIMp27 and HT-29.shCtrl xenografts of nu/nu mice with or without treatment with Dox (1 mg/ml supplemented with 10 mg/ml sucrose in drinking water) and/or oxaliplatin (5 mg/kg by intraperitoneal injection, twice a week). Data are mean ± s.d. or representatives; n = 6 mice per group, one-way ANOVA followed by Tukey's multiple comparison test. Scale bar, 25 μm.

Table S1. Summary of clinicopathological characteristics of the cohort of 77 COAD patients

[1]RS: Reactive score

Characteristics	Case	LIMp27 abundance in COAD (RS[1])	*P* value[2]
**Gender**			
Male	43	6.92± 0.10 [(3)]	0.144
Female	34	5± 0.74	
**Age**			
≥68[(4)]	40	5.07 ± 0.71	0.109
<68	37	7.16 ± 1.11	
**TNM stage**			
I/II	51	6.63 ± 0.81	0.234
III/IV	26	4.98 ± 1.09	
**Histological Grade**			
I/II	59	6.66 ± 0.79	0.107
II-III/III	18	4.17 ± 0.94	

[2]Two-tailed Student's *t*-test; a *P* value less than 0.05 was considered statistically significant

[3]Data shown are mean ± s.e.m.

[4]The median age of the patients in this cohort was 68

Table S2. Summary of proteins that commonly interact with LIMp27 in HT-29 and WiDr cells detected using mass spectrometry

Gene Symbol	MW [kDa]	HT-29		WiDr	
		Coverage [%]	Score Sequest HT	Coverage [%]	Score Sequest HT
HNRNPA0	30.8	28	20.11	41	9.61
KRT10	58.8	21	7.63	32	8.77
MYH2	222.9	4	6.39	2	4.21
KRT1	66	21	5.67	28	24.88
FAM149B1	64.6	2	4.27	3	2.41
THAP5	45.4	2	3.51	2	6.88
ALB	69.3	6	2.42	19	2.09
ODF2	95.3	10	2.29	8	2.08
TUBA3D	49.9	4	2.17	7	2.11
PRSS3	32.5	4	2.15	4	2.04
IREB2	105	2	2.09	2	1.9
KIF20B	210.5	2	1.97	1	1.97
KRT2	65.4	8	1.74	13	4.81

Table S3. List of antibodies

**Antibody**	**Catalogue No.**	**Company**
E2F1 Monoclonal Antibody (KH95)	32-1400	Thermo Fisher Scientific (Waltham, MA)
Monoclonal ANTI-FLAG® M2 antibody produced in mouse	F3165	Sigma-Aldrich (St. Louis, MO)
p21 Waf1/Cip1 (12D1) Rabbit mAb	#2947	Cell Signalling Technology (Beverly, MA)
p27 antibody (F-8)	sc-1641	Santa Cruz Biotechnology (Santa Cruz, CA)
GAPDH antibody (6C5)	sc-32233	Santa Cruz Biotechnology (Santa Cruz, CA)
Lamin A/C Antibody	#2032	Cell Signalling Technology (Beverly, MA)
Goat anti-Rabbit IgG(H+L) Secondary antibody, Alexa Fluor 488	A-11008	Thermo Fisher Scientific (Waltham, MA)
Anti-Histone H3 (tri methyl K4) antibody - ChIP Grade	ab8580	Abcam (Cambridge, United Kingdom)
Anti-Histone H3 (tri methyl K27) antibody [mAbcam 6002] - ChIP G	ab6002	Abcam (Cambridge, United Kingdom)
HNRNPA0 Antibody	NBP2-22293	Novus Biologicals (Littleton, CO)
hnRNP A0 Antibody	#4046	Cell Signalling Technology (Beverly, MA)
Recombinant Anti-p27 KIP 1 antibody [Y236]	ab32034	Abcam (Cambridge, United Kingdom)
hnRNP M Antibody	#28699	Cell Signalling Technology (Beverly, MA)
p53 Antibody	#9282	Cell Signalling Technology (Beverly, MA)
Ki-67 (D3B5) Rabbit mAb	#9129	Cell Signalling Technology (Beverly, MA)

Table S4. List of Reagents

Reagent	Catalogue No.	Company
Cycloheximide	100183	MP Biomedicals (Santa Ana, CA)
Oxaliplatin	O9512	Sigma-Aldrich (Saint Louis, MO)
Actinomycin D	50-76-0	Sigma-Aldrich (Saint Louis, MO)
SlowFade™ Gold Antifade Mountant	S36936	Thermo Fisher Scientific (Waltham, MA)
SUPERase•In™ RNase Inhibitor (20 U/µL)	AM2694	Thermo Fisher Scientific (Waltham, MA)
Recombinant Proteinase K Solution (20 mg/mL)	AM2548	Thermo Fisher Scientific (Waltham, MA)
20× SSC	AM9765	Thermo Fisher Scientific (Waltham, MA)
Pierce™ Anti-DYKDDDDK Magnetic Agarose	A36797	Thermo Fisher Scientific (Waltham, MA)
Pierce™ 3x DYKDDDDK Peptide	A36805	Thermo Fisher Scientific (Waltham, MA)
RiboLock RNase Inhibitor (40 U/µL)	EO0382	Thermo Fisher Scientific (Waltham, MA)
Halt™ Protease and Phosphatase Inhibitor Cocktail (100×)	78440	Thermo Fisher Scientific (Waltham, MA)
Glutaraldehyde solution	G5882-10X1ML	Sigma-Aldrich (Saint Louis, MO)
PMSF	78830-1G	Sigma-Aldrich (Saint Louis, MO)
Doxycycline	D9891	Sigma-Aldrich (Saint Louis, MO)
BrdU Cell Proliferation Assay Kit	6813	Cell Signaling Technology (Danvers, MA)

Table S5. siRNA, shRNA, and sgRNA sequences

si/sh/sgRNA	5'-3'
Ctrl siRNA	UUCUCCGAACGUGUCACGUTT
LIMp27 siRNA-1	CUGCCUAACAGAGAACAAUTT
LIMp27 siRNA-2	CAGUGGAGGACAGGAAUAUTT
p27 siRNA-1	GCGCAAGUGGAAUUUCGAUUUTT
p27 siRNA-2	CCUGCAACCGACGAUUCUUTT
E2F1 siRNA-1	CCUGGAAACUGACCAUCAGTT
E2F1 siRNA-2	GCUGGACCACCUGAUGAAUTT
p53 siRNA-1	CGGCGCACAGAGGAAGAGAAUCUCTT
p53 siRNA-2	CUACUUCCUGAAAACAACGTT
p21 siRNA-1	GAUGGAACUUCGACUUUGUTT
p21 siRNA-2	GCAUGACAGAUUUCUACCATT
hnRNPA0 siRNA-1	CUUUGUCGGAGGCCUUAAATT
hnRNPA0 siRNA-2	CCGAGAUUAUUGCCGACAATT
LIMp27 shRNA-1 sense	TCCCCTGCCTAACAGAGAACAATTTCAAGAGAATTG TTCTCTGTTAGGCAGTTTTTC
LIMp27 shRNA-1 antisense	TCGAGAAAAACTGCCTAACAGAGAACAATTCTCTTG AAATTGTTCTCTGTTAGGCAG
LIMp27 shRNA-2 sense	TCCCCAGTGGAGGACAGGAATATTTCAAGAGAATA TTCCTGTCCTCCACTGTTTTTC
LIMp27 shRNA-2 antisense	TCGAGAAAAACAGTGGAGGACAGGAATATTCTCTT GAAATATTCCTGTCCTCCACTG
Ctrl shRNA sense	TCCCTTCTCCGAACGTGTCACGTTTCAAGAGAACGT GACACGTTCGGAGAATTTTTC
Ctrl shRNA antisense	TCGAGAAAAATTCTCCGAACGTGTCACGTTCTCTTG AAACGTGACACGTTCGGAGAA
hnRNPA0 shRNA sense	CTAGCCTTTGTCGGAGGCCTTAAATACTAGTTTTAA GGCCTCCGACAAACTTTTTG
hnRNPA0 shRNA antisense	AATTCAAAAACTTTGTCGGAGGCCTTAAAACTAGTA TTTAAGGCCTCCGACAAACG
LIMp27 AUE sgRNA sense	CACCGACATCTCACCAATTTTAAAT
LIMp27 AUE sgRNA antisense	AAACATTTAAAATTGGTGAGATGTC

Table S6. List of primers for qPCR and ChIP

Primers	Sequences (5'-3')
LIMp27-F	GATGCCCAGCTTTGGATAA
LIMp27-R	CAGTTACTTCAGGCCATCTG
LINC01357-F	GCTCCTGGTGGGTATTAAAG
LINC01357-R	GGTCAGGTATCTGGGTTCTA
E2F1 CHIP--621F	CCCTCTTACCTGCTCTTCTTTC
E2F1 CHIP--522R	GGGAGAACCCATGCAACTT
E2F1 CHIP--4236F	ACCGGAGCAAGTTGCCACTG
E2F1 CHIP--4333R	ACAGACCACTTGGCTCTACC
PLANE-F	TACATACAGTGACCCAAAGAGCA
PLANE-R	CAGTGCTTCTGAACGCCTCTT
CDKN1B promoter ChIP-F	CCGCAACCAATGGATCTC
CDKN1B promoter ChIP-R	GTGGAAGGGAGGCTGAC
qPCR-RPL13A-F	CCTGGAGGAGAAGAGGAAAGAGA
qPCR-RPL13A-R	TTGAGGACCTCTGTGTATTTGTCAA
qPCR-CDKN1B-F	GCTAACTCTGAGGACACGCATT
qPCR-CDKN1B-R	GCTCCACAGAACCGGCATT
qPCR-CDKN1B-3UTR-F	AGCAACAGAAACCTATCCTCAC
qPCR-CDKN1B-3UTR-F	CATTCAAAACTCCCAAGCACC
qPCR-CDKN1A-F	CCAGCATGACAGATTTCTACCAC
qPCR-CDKN1A-R	GACACACAAACTGAGACTAAGGCA
U6-F	GCTTCGGCAGCACATAT ACTAAAAT
U6-R	CGCTTCACGAATTTGCGTGTCAT
hnRNPA0-F	GCTTTGGCTTCGTGACCTAC
hnRNPA0-R	AACTGCGAGAAGTGCTCGAT
LIMp27 ΔhnRNPA0-BR-genotyping F	ACGCGAATGAAATTTGGTGCCATCACTCG
LIMp27 ΔhnRNPA0-BR-genotyping R	GGAGAGATTGAAAGATGGCACCAAGATTG

Table S7. List of probes for RNA pulldown

Probes	Sequences (5'-3')
LIMp27 Probe AS1	GGGGATTGATCTCCCAAGGG
LIMp27 Probe S1	CCCTTGGGAGATCAATCCCC
LIMp27 Probe AS2	TTCTTCAGTTACTTCAGGC
LIMp27 Probe S2	GCCTGAAGTAACTGAAGAA
LIMp27 Probe AS3	ATTGTTCTCTGTTAGGCAG
LIMp27 Probe S3	CTGCCTAACAGAGAACAAT
p27 probe AS1	TTAGACACTCGCACGTTTGA
p27 probe S1	TCAAACGTGCGAGTGTCTAA
p27 probe AS2	TTTGGGGAACCGTCTGAAAC
p27 probe S2	GTTTCAGACGGTTCCCCAAA
p27 probe AS3	ATCACCATTCTGCTGAGTAA
p27 probe S3	TTACTCAGCAGAATGGTGAT
